# Effectiveness of a Mind–Body Intervention at Improving Mental Health and Performance Among Career Firefighters

**DOI:** 10.3390/ijerph22081227

**Published:** 2025-08-06

**Authors:** Anthony C. Santos, Seth Long, Christopher P. Moreno, Dierdra Bycura

**Affiliations:** 1 Department of Health Sciences, Northern Arizona University, Flagstaff, AZ 86011, USA; christopher.moreno@nau.edu (C.P.M.); dierdra.bycura@nau.edu (D.B.); 2 Department of Physical Therapy and Athletic Training, Northern Arizona University, Flagstaff, AZ 86011, USA; seth.long@nau.edu

**Keywords:** firefighters, mental health, performance, functional training, psychological resilience, intervention, longitudinal research

## Abstract

Almost one in three firefighters develop mental health disorders at some point during their careers, a rate double that in the general population. Frequent exposures to potentially traumatic situations can contribute to symptoms of these disorders, two of the most common being depression and post-traumatic stress disorder (PTSD). While various psychological interventions have been implemented among this group, reports of their effectiveness include mixed results. To this end, the current study endeavored to test the effectiveness of a 12-week intervention combining occupationally-tailored high-intensity functional training (HIFT) and psychological resilience training (RES) in reducing depressive and post-traumatic stress symptoms (PTSSs), as well as increasing psychological resilience and mental wellbeing, in career firefighters. Thirty career firefighters completed four mental health measurements over 17 weeks while anthropometrics and physical performance (i.e., number of stations completed in 20 min during an eight-station simulated job-task circuit workout [T-CAC]) were measured pre- and post-intervention. Pre to post comparisons were made via repeated-measures *t*-tests. Significant mean differences were observed for T-CAC stations completed, PTSSs, and psychological resilience between pre- and post-intervention. In future interventions, researchers should actively engage firefighters, maximize integration with daily operations, and employ culturally-relevant practices to explore the links between physical and mental health.

## 1. Introduction

By nature, the firefighting profession exposes career and volunteer firefighters to a multitude of potentially dangerous, threatening, and traumatic situations in the pursuit of saving lives and property. These frequent exposures require a great deal of physical fitness and psychological resilience and, over time, can lead to lasting effects on one’s body and mind. Almost one in three firefighters develop mental health disorders at some point during their careers, a rate double that in the general population [1,2,3]. However, frequent exposures to potentially traumatic events, environmental hazards, and disturbed sleep patterns can contribute to symptoms of mental health disorders, two of the most common being depression and post-traumatic stress disorder (PTSD) [1,2,4,5,6,7,8,9]. Overall, seminal research indicates that 11% of firefighters suffer from depression [10], with more contemporary research suggesting similar results (i.e., ~10%) [11].

Previous stressful life events like traumas or personnel losses can also increase firefighters’ risks of experiencing mental health issues following a disaster response or other major event while on-duty [1,10]. As a result of such traumas, firefighters can experience post-traumatic stress symptoms (PTSSs). While PTSD is a recognized medical condition by the American Psychiatric Association (APA), as reported in the Diagnostic and Statistical Manual of Mental Disorders, 5th edition (DSM-5), PTSSs reflect sub-clinical symptoms of the diagnosable disorder of PTSD. Further, although the presence of PTSSs (e.g., hyperarousal, vivid flashbacks, involuntary intrusive thoughts or recollections, nightmares, and numbing and/or avoidance of triggering activities relating to memories of traumatic events) [2,12,13,14] may or may not develop into PTSD, these symptoms can still negatively impact a person’s mental health.

### 1.1. Exercise and Mental Health

Structured exercise can elicit various positive effects on mental health and psychological wellbeing in all individuals, including firefighters. Seminal and contemporary research has acknowledged exercise as therapeutically effective with similar effectiveness to some psychotherapies and antidepressant medications [15,16,17,18,19,20,21,22,23] for treating depression, as well as reducing and even preventing depressive symptoms among clinical and non-clinical populations, regardless of age, sex, or geographical region [15,16,17,18,19,20,21,22,23,24,25,26,27,28,29,30]. Similar positive effects from exercise have been shown to decrease anxiety and PTSSs, namely through improved sleep quality, quality of life (QOL), self-esteem, self-efficacy, mood, and physical wellbeing [26,27,31,32,33,34,35,36,37].

More specific to firefighters, strong evidence exists supporting exercise as both a preventative measure and adjunct treatment for commonly experienced mental disorders (i.e., depression, anxiety, and PTSD) that also provides various ancillary physical health benefits [24,28,35]. Regular physical activity can also reduce alcohol dependence among firefighters and is associated with a 17% reduction in incident cases of depression [35], as well as anxiety and stress-related disorders more generally [37]. A meta-review by Ashdown-Franks et al. [37] states that both aerobic exercise (AE) and combined AE and strength training at moderate to vigorous-intensity can significantly reduce PTSSs compared to control conditions (i.e., usual care or wait-list controls; Hedges’ *g* = −0.31, 95% CI [−0.60, −0.02]) [38]. In this case, they and Rosenbaum et al. [38] list usual care as trauma-focused cognitive behavioral therapy (CBT), eye-movement desensitization and reprocessing (EMDR), and pharmacotherapy (i.e., SSRIs). Rosenbaum et al. also reported preliminary findings that mental health interventions that include exercise can result in a significantly greater reduction in depressive symptoms in those with PTSD than controls (Hedges’ *g* = −0.37, 95% CI [−0.69, −0.05]) [37,38].

As a whole, exercise can improve mental wellbeing and is a viable preventative and/or adjunctive option for improving various mental health outcomes. Occupationally-specific high-intensity functional training (HIFT) has the potential to positively affect both physical and psychological factors in firefighters [23,29,30,37]. HIFT emphasizes functional, multi-joint movements performed as both aerobic and strength-training exercises that elicit increased muscle recruitment to induce greater improvements to cardiorespiratory endurance, muscular strength, and flexibility than either aerobic or strength training alone [39,40]. HIFT has also been positively correlated with participant enjoyment, social support, and intrinsic motivation and can serve as a potent training stimulus for firefighters to improve operational readiness alongside overall fitness [39,41,42,43]. Given the demonstrated beneficial effects of exercise on physical and mental health, exercise has the potential to elicit positive effects on mental health and psychological wellbeing in firefighters.

### 1.2. Psychological Resilience and Mental Health

The prevalence and risks for depression and PTSD in this population necessitate further research regarding the promotion and protection of firefighters’ mental health. To these ends, researchers advocate for the facilitation of psychological resilience for the prevention of PTSD and reduction of depressive symptoms [44,45,46]. The literature also supports exercise as a means to positively affect physical and psychological resilience, specifically by increasing mood, self-esteem, sleep quality, QOL, and physical wellbeing [31,32,33,47].

There is a need to directly bolster psychological resilience in firefighters through effective and comprehensive means [48,49,50,51,52,53,54,55,56]. The literature indicates that resilience, defined as one’s ability to successfully adapt to stressors and maintain psychological wellbeing in the face of adversity [47], is a primary protective factor against a multitude of mental health issues, including depression and PTSD [1,57,58,59,60]. In this regard, psychological resilience training (RES) has the capacity to improve mental health and wellbeing in firefighters, specifically pertaining to PTSSs. To date, RES programs implemented with firefighters have been shown to successfully increase mood and emotion regulation skills [47,61], help-seeking behaviors [49], QOL [62], and mental wellbeing [57]. Firefighters who can draw on resilience as a personal resource can also better resist hazards associated with traumatic situations and better cope with post-traumatic stress [63,64].

Finally, the literature also suggests the potential of combined approaches for improving firefighters’ psychological functioning. In terms of reducing depressive symptoms, exercise has been demonstrated as an effective, low-cost adjunct to psychological interventions employing cognitive reappraisal (i.e., one’s ability to reevaluate and reframe experiences in a more positive light) [47], active emotional coping, arousal management, and cognitive monitoring techniques (i.e., RES) [47,55]. Concerning PTSSs, moderate- to high-intensity exercise has also been associated with reduced PTSS severity over time via improved sleep quality, mood, autonomy, and self-esteem [30,31,32,33,65,66]. Also, because many pharmacological and psychotherapeutic approaches fail to achieve full remission in all individuals, structured exercise as a non-stigmatizing, low-cost option could potentially improve long-term outcomes across a range of mental health disorders [37]. Combined with the positive effects of RES on depression, stress, anxiety, vigor, and QOL [49,67,68], moderate- to high-intensity exercise could significantly improve wellbeing in addition to physical and mental preparedness in firefighters.

To date, previous studies investigating mental health in this population have identified the increasing prevalence of depression, anxiety, and stress-related conditions, although few studies have attempted an intervention to help address these mental health concerns among career firefighters.

The purpose of this study was to explore the impact of an occupationally-tailored 12-week intervention combining HIFT and RES on mental health (i.e., depressive symptoms, post-traumatic stress symptoms, psychological resilience, and mental wellbeing) in career firefighters. The primary aim of the study was to observe change over time for each of the four mental health outcome variables in a group of 30 career firefighters.

Additionally, the study intervention was designed to specifically address the National Fallen Firefighters’ Foundation (NFFF) research agenda toward firefighter health and safety, with a special emphasis on behavioral health to address firefighters’ concerns with post-traumatic stress, depression, suicide, and related issues.

## 2. Materials and Methods

A longitudinal, one-group, double pre-test quasi-experiment was implemented to test the effectiveness of a combined exercise and psychological resilience training intervention at improving mental health (i.e., PTSSs, depressive symptoms, mental wellbeing, and psychological resilience) among firefighters over 17 weeks. Components of the intervention included occupation-specific HIFT prescribed in a daily undulating periodized fashion.

Additionally, we incorporated The Road to Resilience (R2R) program by The First Twenty^®^ (TF20; Narberth, PA, USA). R2R is an occupationally-tailored, web-based health and wellness program designed by firefighters for firefighters with the help of experts in the fields of behavioral and physical health [69,70,71]. This program was selected to better impact mental wellbeing and physical performance outcomes among this group, as well as facilitate increased access and scalability of mental health interventions among career firefighters.

**Figure 1:**
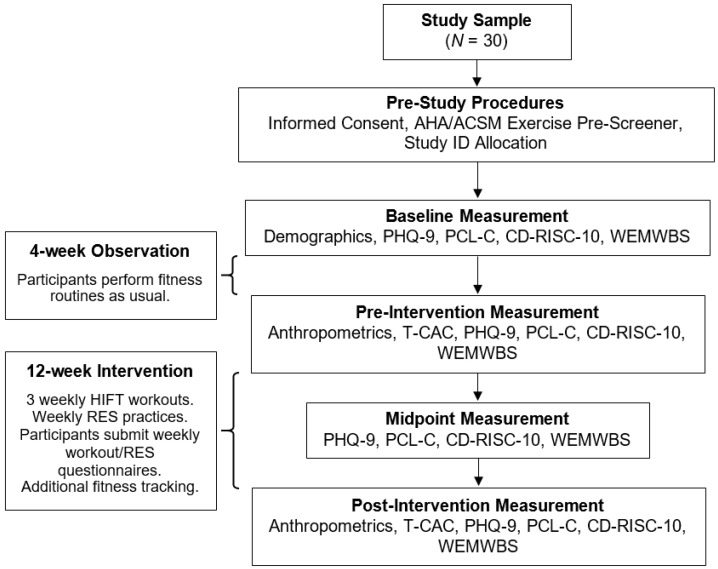
One-group longitudinal study design with double pre-test. AHA, American Heart Association; ACSM, American College of Sports Medicine; CD-RISC-10, 10-item Connor–Davidson Resilience Scale; HIFT, high-intensity functional training; PCL-C, PTSD Checklist—Civilian version; PHQ-9, 9-item Patient Health Questionnaire; RES, resilience training; T-CAC, Time-Critical Athlete Challenge; WEMWBS, 14-item Warwick–Edinburgh Mental Wellbeing Scale.

Participants completed four mental health outcome measurements during the full study period (i.e., baseline [study week 0], pre-intervention [study week 4], midpoint [study week 11], and post-intervention [study week 17]). The survey measurements that occurred before the intervention period (i.e., baseline and pre-intervention time points) were used to identify possible fluctuations in participants’ mental health prior to the introduction of any intervention practices. Without the inclusion of participant randomization into treatment and control groups, this double pre-test design was chosen for its propensity to reduce the plausibility of maturation and regression threats to internal validity. A full layout of major study events can be found below in Figure 1.

All survey data were collected and managed using Research Electronic Data Capture (REDCap) [72,73]. REDCap is a secure, web-based software platform designed to support validated data capture for research studies. All study protocols, from design to implementation to write-up, were approved by the Institutional Review Board (IRB; Project Number 1792624-1).

### 2.1. Participants and Exercise Pre-Screening

Career firefighters of all genders and ethnicities aged 18–55 years were recruited from three fire departments in the southwest United States. Recruitment methods included emails inviting participants to attend Zoom presentations provided by study personnel explaining the TF20 platform, study overview, and intervention requirements, after which any and all questions were answered.

At the conclusion of the recruitment presentation, a QR code was provided on screen linking potential participants to an online informed consent form where first and last names, electronic signatures, and email addresses were provided if the participants were interested in study inclusion. Once informed consent was provided, potential participants were immediately sent an exercise prescreening form where health status; cardiovascular (CV) risk factors, events, and symptoms; other health issues; and overall exercise readiness were assessed.

The American Heart Association (AHA)/American College of Sports Medicine (ACSM) Exercise Pre-Screener was used to screen out potential participants in this regard. Only participants who were rated healthy enough for moderate-to-vigorous physical activity as indicated by the prescreening form were selected for study inclusion. Those with risk factors or history of CV events were contacted for further information. It should also be noted that career firefighters who are cleared for active duty (i.e., all study participants) undergo routine medical evaluations and are ultimately cleared for strenuous activity (e.g., fireground operations or exercise) by department administrators and medical personnel.

Participants were also excluded from the study if they were pregnant or became pregnant or were unable to safely perform exercises and/or otherwise participate in the study due to injury or health issues. Overall, 30 participants were recruited (3 female), representing 12 independent fire stations.

### 2.2. Pre- and Post-Intervention Procedures

Prior to the intervention start date, participants’ demographics, anthropometrics (i.e., height, weight, body mass index [BMI], lean body mass [LBM], and body fat percentage [%BF]), and physical performance abilities were measured. Anthropometrics were measured via bioelectrical impedance analysis (BIA) using an InBody 570 Body Composition Analyzer (InBody; Seoul, Republic of Korea). Firefighter-specific physical performance testing was also measured across an eight-station circuit workout (the Time-Critical Athlete Challenge [T-CAC]), in which participants completed as many rounds as possible under a 20 min time cap.

The T-CAC was designed to simulate the Candidate Physical Ability Test (CPAT), the gold-standard firefighter physical ability test designed and validated by the International Association of Fire Fighters’ (IAFF) and International Association of Fire Chiefs’ (IAFC) Joint Labor Management Wellness-Fitness Initiative (WFI) Task Force [74]. The T-CAC workout was performed immediately pre- and post-intervention at the local Training Facility using available firefighting equipment, facilities, and personnel. Firefighters completed the workout in full turnout gear, including boots, bunker coat, pants, helmet, work gloves, and empty air bottle (without face mask), after a course walkthrough immediately following body composition analysis.

### 2.3. Mental Health Outcome Measures

Four psychological instruments were used to measure mental health outcomes of depressive (Patient Health Questionnaire [PHQ-9]) [75] and post-traumatic stress symptoms (PTSD Checklist, Civilian version [PCL-C]) [76], as well as psychological resilience (Connor–Davidson Resilience Scale [CD-RISC10]) [77] and mental wellbeing (Warwick–Edinburgh Mental Wellbeing Scale [WEMWBS]) [78]. The PHQ-9 is a 9-item instrument with questions answered in relation to presence of bothersome symptoms over the past two weeks, ranging from not at all (0), several days (1), more than half the days (2), to nearly every day (3). Scores can range from 0 to 27. Clinical cutoff scores for depressive symptom severity were set as minimal (0–4), mild (5–9), moderate (10–14), moderately severe (15–19), and severe (20–27) [79]. An initial validation study indicated good agreement between PHQ-9 diagnosis and independent diagnosis by mental health professionals (*κ* = 0.65), with overall accuracy of 85%, sensitivity of 75%, and specificity of 90% [75]. A score ≥10 corresponds with a sensitivity of 88% and a specificity of 88% for major depression when compared to criterion standard mental health professional interviews. The instrument also has excellent internal reliability at *α* = 0.89 and 48 h test–retest reliability at *r* = 0.84 [79].

The PCL-C is a 17-item self-report measure based on the DSM-IV’s 17 symptoms for post-traumatic stress disorder in traumatized populations. Questions are rated on a 5-point Likert-type scale to the degree that symptoms have been bothersome over the past month, ranging from not at all (1), a little bit (2), moderately (3), quite a bit (4), to extremely (5). Scores can range from 17 to 85 [76]. An initial validation study demonstrated *κ* = 0.64 for PTSD diagnosis, test–retest reliability of *r* = 0.96, and internal consistency of *α* = 0.94 [76]. Depending on the specific cutoff score used, sensitivity ranged between 0.78 and 0.94, and specificity between 0.68 and 0.71, with comparable values found between this measure and other accepted self-report PTSD measures [80]. For the current study, a cutoff score of 44 was used as a measure of probable PTSD diagnosis, as this was previously found to maximize diagnostic efficiency by Blanchard et al. [76]. Although a more current version of the Checklist exists (i.e., PCL-5), the PCL-C was utilized in the current study to reflect past practices in research concerning PTSSs and PTSD in firefighters. Additionally, a study by Moshier et al. [81] comparing PCL-C and PCL-5 scores suggested a great deal of overlap (*r* = 0.95) between the measures, and authors provide a “crosswalk” with which to convert scores between the two. The PCL-C also has three less questions than the PCL-5 (i.e., 17 versus 20), and with three other instruments being administered to participants at each measurement time point, a measure of similar validity and fewer items was chosen.

The CD-RISC10 is perhaps the most widely administered resilience scale among various US and international military, first responder, and general populations [82] and was used to measure psychological resilience. An initial validation study rated the measure’s internal consistency at *α* = 0.85 [77], and subsequent examinations indicated test–retest reliability between 0.78 and 0.90 [83]. The 10 scale items are rated on a 5-point Likert-type scale ranging from not true at all (0), rarely true (1), sometimes true (2), often true (3), to true nearly all of the time (4). Scores can range from 0 to 40. In general, higher CD-RISC10 scores correspond with increased psychological resilience. Authors report that a score in the first (scores between 0 and 29) or second (scores between 30 and 32) quartiles may suggest difficulties in one’s ability to cope with stress or bounce back from adversity [84] (p. 3). Items relate to cognitive flexibility, self-efficacy, ability to regulate emotion, optimism, cognitive focus, and maintaining attention under stress [84]. The CD-RISC10 was developed by Campbell-Sills and Stein’s work relating to the treatment, assessment, diagnosis, biological characterization, cross-cultural study, epidemiology, and risk factors for PTSD [85]. Since the current study aimed to reduce PTSSs over time in firefighters by bolstering psychological resilience, this measure was chosen to better qualify any changes in PTSSs that were observed over the study period.

Lastly, the WEMWBS is a comprehensive 14-item instrument designed to measure mental wellbeing at the population level and intended for mental health promotion initiatives [78]. Scale items are rated on a 5-point Likert-type scale ranging from none of the time (1), rarely (2), some of the time (3), often (4), to all of the time (5). Scores can range from 14 to 70. In general, higher scores on the WEMWBS correspond with increased mental wellbeing. The scale initially displayed a Cronbach’s *α* = 0.91, test–retest reliability of *r* = 0.83 at one week, and lesser social desirability bias to similar instruments when tested among university students and representative population samples in the UK [78,86]. The WEMWBS was further validated and shown to be sensitive to change in groups and individuals across five studies in 2012, with Cronbach’s *α* ≥ 0.86 [87]. The scale focuses on Positive Psychology concepts and expanded perspectives of mental wellbeing, namely from a combined hedonic (i.e., encompassing one’s subjective experience of happiness and general life satisfaction) and eudaimonic perspective (i.e., including one’s positive psychological functioning, mutually beneficial relationships with others, and self-realization) [88]. More generally, these two overarching perspectives relate to feeling good and functioning well, respectively. While the WEMWBS was not designed as a screener for depression or broader mental illness, authors do suggest that a score of 40 or below could indicate high risk of major depression, while scores between 41 and 45 suggest high risk of psychological distress [88].

These scales were employed at four different time points over the study timeline in order to better observe possible changes in participants’ mental health over time. These measurements occurred during study weeks 0, 4, 11, and 17 as the baseline, pre-intervention, midpoint, and post-intervention measurement time points, respectively. Overall, each survey battery started with a Survey Welcome Page on which participants were reminded of general confidentiality; provided with contact information of study PIs; and provided with tables listing local, county, state, and national mental health resources. These resources and PI contact info were also provided to participants on each survey battery’s Departure Page.

### 2.4. Intervention Protocols

Each intervention week included three HIFT workouts lasting at least 20 min each, as well as online RES modules for a total weekly time requirement of 1.5–2 h. Weekly resilience training modules consisted of four primary practices. Namely, these practices included a weekly Expeditionary Readiness Challenge, Visualization & Positive Self-talk practice, weekly Mantra practice, and weekly “Breathe with your Mantra” practice (i.e., four-second box breathing while repeating the mantra to oneself). The emphasis of HIFT workouts changed daily, and the workouts were periodized in a undulating fashion; specifically, the volumes and intensities of each workout fluctuated widely throughout each intervention week [89] but slowly progressed toward higher intensities over time. Overall, HIFT workouts were provided to participants via email on preformatted workout cards on Mondays of each intervention week. TF20’s Road to Resilience Weekly Challenge & Mantra were emailed to participants on Sundays of each intervention week, while subsequent TF20 Road to Resilience Challenge Check-In messages were sent on Tuesdays, and a final TF20 Road to Resilience Mantra Check-In was sent on Thursdays.

Short videos were also provided to participants demonstrating safe performance of most exercises. Overall, 169 videos totaling 173 min and 8 s of content were created. Exercise modifications, progressions, and regressions were also provided to participants here in order to accommodate fitness abilities and common joint injuries (e.g., to shoulders, lower-back, knees) associated with this population.

Additional fitness tracking was also collected weekly through the Workout and RES questionnaire to account for any exercise performed outside our three prescribed weekly HIFT workouts. Namely, participants were asked if they performed any additional workouts during the week, and if they answered “Yes,” a subsequent question concerning the number of additional workouts appeared. Based on the whole number they entered here, follow-up questions regarding the amount of time they spent exercising, the intensity of their exercise (i.e., recorded as a rating of perceived exertion [RPE] value), and type of exercise they performed were asked. If they answered “No” to performing additional workouts that week, the questionnaire ended there. These weekly questionnaires of intervention practices were used to track participants’ adherence to intervention protocols. That is, completed questionnaires were equated with completed workouts and RES practices. If participants did not complete a workout or RES practice, they were instructed to leave the questionnaire blank.

### 2.5. Statistical Analyses

Participant-specific survey batteries were distributed via email and were only accessible to those included in the study. All survey responses and metadata were collected using Research Electronic Data Capture (REDCap) [72,73]. All data remained deidentified, confidential, password protected, and stored on a secure University server. Microsoft Excel [90] was also used to track and run basic descriptive statistics (e.g., means, medians, standard deviations) on intervention adherence, as well as workout and R2R ratings. IBM SPSS Statistics (Version 28) [91] was also utilized to generate difference scores, total adherence calculations, repeated-measures *t*-tests, independent-samples *t*-tests, Hedges’ *g* effect sizes, and boxplots. Repeated-measures *t*-tests were performed to assess mean differences between baseline and pre-intervention measurements (i.e., study week 0 to 4), as well as pre-intervention and post-intervention measurements (i.e., study week 4 to 17).

Repeated-measures *t*-tests employed pairwise exclusion, and participants with missing data (i.e., incomplete data “pairs” from repeated measurements) were not included in models. Further, incomplete mental health questionnaires were counted as missing, and associated scores were not entered into statistical models. Also, outliers were not removed from these tests in order to preserve degrees of freedom and make use of the full sample data. The influence of outliers will be discussed in Section 3.6, Mental Health Outcome Measurement Distributions. 

Hedges’ *g* values can be interpreted as mean differences in standard deviations, in that a value of *g* = 1.00 represents a mean difference of one standard deviation, *g* = 2.00 represents a mean difference of two standard deviations, and so on [92]. While suggested small, medium, and large ranges of effect size vary considerably among research fields [93,94,95], sources still recommend using Cohen’s values of 0.20, 0.50, and 0.80 as small, medium, and large effect sizes, respectively [93,96,97]. Post hoc sample size and power analyses were completed using G*Power 3.1.9.4 [98,99] for achieved power, given α, sample size, and effect size, for repeated-measures *t*-test results. Statistical significance was set as two-tailed *p*-values ≤ 0.05, and all 95% CIs, effect sizes, and ranges are reported in full. Multiple comparisons were also controlled for using Bonferroni corrections [100] where appropriate.

SAS software, Version 9.4, of the SAS System for Windows (SAS Institute Inc.) [101] was used to calculate intraclass correlation coefficients (ICCs) and perform multilevel modeling (MLM). Analyses were conducted using two-level models with random intercepts including between-subject (i.e., participant at level 2) effects and time (i.e., measurement occasion at level 1) for outcome variables. Maximum likelihood (ML) estimation was chosen as the estimation method for variance components to maximize the likelihood of describing the full sample data while treating the fixed effects as known [102,103]. An unstructured covariance matrix and the between–within method for denominator degrees of freedom were also utilized. In all reported models, *B* coefficients for time (i.e., fixed effects for growth) reflect change in each outcome over one-week increments of time during the intervention period.

MLMs were centered on the pre-intervention measurement occasion, rather than post-intervention, due to loss to follow-up for anthropometrics and job-task performance post-testing, as well as corresponding losses in degrees of freedom. Outliers were similarly not removed from these models in order to preserve degrees of freedom. In all growth models (i.e., those modeling the effects of time), growth was calculated as the difference between measurement occasions in weeks for each model outcome variable. Random effects, model deviance values (i.e., −2 log-likelihood test differences, Akaike Information Criterion [AIC], and Bayesian Information Criterion [BIC]), and pseudo-*R*^2^ values are also presented with each model. Pseudo-*R*^2^ values were calculated using the “FitStatistics” output and PROC REG in SAS [101] for each growth model to gauge model fit over each original unconditional model for the means. Overall, MLMs estimated the effects of demographics, intervention adherence, and relevant covariates on mental health outcomes, anthropometrics, and simulated job-task performance.

For all outcomes, Model 1 presents results for unconditional models for the means, in which no predictors were included and the fixed effects reflect outcome grand mean values at pre-intervention. Model 2 presents results for unconditional growth models that indicate an omnibus presence or lack of change in outcome values over time without any covariates. The first conditional models are presented as Model 3, in which mean-centered baseline scores of each outcome variable were included as covariates alongside growth (i.e., time in weeks between measurement occasions).

When examining main effects of demographics on outcomes at pre-intervention (week 4), categorical variables representing participants’ relationship status, education level, fire department rank, and race/ethnicity were dichotomized due to differences in group sizes and ease of interpretation. Overall,

- Model 4 (Age_MC_) controlled for participants’ mean-centered age at 39 years (*M* = 39.70 years, *SD* = 7.62, range = 23.00–57.00);
- Model 5 controlled for level of education, dichotomized into 0 (Some college but no degree [*n* = 2] or Associate degree [*n* = 13]) and 1 (Bachelor degree [*n* = 13] or Graduate degree [*n* = 2];
- Model 6 controlled for fire department rank, dichotomized into 0 (Firefighter [*n* = 8] or Engineer [*n* = 6]) and 1 (Captain [*n* = 10] or Battalion Chief [*n* = 6]);
- Model 7 (Years of service_MC_) controlled for years of service mean-centered at 15 (*M* = 15.43 years, *SD* = 8.27, range = 1.00–35.00);
- Model 8 controlled for participants’ combined race and ethnicity, dichotomized into 0 (Race: White [*n* = 1], Other [*n* = 1], Don’t know [*n* = 1], or Prefer not to say [*n* = 1]; Ethnicity: Hispanic [*n* = 3] or Prefer not to say [*n* = 1]) and 1 (Race: White [*n* = 26]; Ethnicity: Not Hispanic [*n* = 26]);
- Model 9 controlled for relationship status, dichotomized into 0 (Single [*n* = 3], In a relationship [*n* = 2], or Divorced [*n* = 1]) and 1 (Married [*n* = 24]);
- Model 10 controlled for participants’ biological sex as 0 (Male [*n* = 27]) and 1 (Female [*n* = 3]).

Concerning the main effects of intervention adherence and additional fitness tracking on mental health outcomes, MLMs were structured as follows:

- Model 4 (Combined adherence_STD_) controlled for participants’ standardized combined adherence (see Tables S12–S15 for a description of variable standardization);
- Model 5 controlled for Combined adherence_STD_ plus the Combined adherence_STD_ × growth interaction;
- Model 6 controlled for Combined adherence_STD_, the Combined adherence_STD_ × growth interaction, and additional fitness tracking variables (i.e., mean-centered additional weekly workouts, mean-centered additional weekly minutes of exercise, and mean-centered rating of perceived exertion [RPE] of additional workouts);
- Model 7 (HIFT adherence_STD_) controlled for participants’ standardized HIFT workout adherence;
- Model 8 controlled for HIFT adherence_STD_ plus the HIFT adherence_STD_ × growth interaction;
- Model 9 controlled for HIFT adherence_STD_, the HIFT adherence_STD_ × growth interaction, and additional fitness tracking variables;
- Model 10 (RES adherence_STD_) controlled for participants’ standardized RES practice adherence;
- Model 11 controlled for RES adherence_STD_ plus the RES adherence_STD_ × growth interaction;
- Model 12 controlled for RES adherence_STD_, the RES adherence_STD_ × growth interaction, and additional fitness tracking variables.

For MLMs concerning the main effects of intervention adherence and additional fitness tracking on anthropometrics and job-task performance (see Tables S7–S11), all aforementioned tests were conducted excluding pre-intervention values since these variables were only measured twice and all models were already centered on the pre-intervention occasion. Due to the large number of tests performed, the corresponding MLM result sections will only detail statistically significant models for each outcome tested. However, full model summary tables are provided in Supplementary Materials.

## 3. Results

### 3.1. Sample Characteristics

Participants’ demographics are presented in Table 1 below. The study sample consisted of 30 firefighters (3 female) from three fire departments in the southwest United States.

### 3.2. Anthropometric and Job-Task Performance Measurements

Participants’ anthropometrics and T-CAC performance were measured pre- and post-intervention during study weeks 4 and 17, respectively. Overall, all participants completed the pre-intervention anthropometric and T-CAC measurements, while 20 returned for post-intervention measurements (i.e., attrition rate of 33%). The results from each measurement occasion are presented in Table 2 below for the full and returning samples.

#### 3.2.1. Group Differences Between Pre- and Post-Testing Samples

Post hoc independent-samples *t*-tests were performed to identify possible group differences between participants who were present (*n* = 20) and absent (*n* = 10) for anthropometric and T-CAC post-testing. Group differences were tested for adherence (i.e., total HIFT workouts, RES practices, and mental health surveys completed), additional fitness tracking (i.e., additional workouts, minutes of exercise, and RPE intensity of exercise completed outside of prescribed HIFT workouts), and mental health outcome variables (i.e., mean PHQ-9, PCL-C, CD-RISC10, and WEMWBS scores) at post-intervention.

Overall, there were no significant group differences between those present or absent for post-testing for demographics (*p*’s > 0.05); total RES practices completed (*p*’s > 0.05); additional fitness tracking (*p*’s > 0.05); or any mental health outcome variable at any measurement occasion (*p*’s > 0.05). However, group differences were identified for total HIFT workouts completed (*t*(9.25) = -2.68, *p* < 0.05). On average, participants absent from post-testing (*M* = 24.50, *SD* = 7.37) performed 7.50 fewer total workouts (*SE* = 2.80) than participants present for post-testing (*M* = 32.00, *SD* = 4.58). There were 36 total workouts prescribed to participants over the intervention period. Levene’s test indicated unequal variances between groups for this comparison (*F*(1, 26) = 4.25, *p* < 0.05), and the *t*-test for equal variances not assumed is reported. Significant group differences were observed for total mental health surveys completed as well. Participants absent from post-testing (*M* = 14.40, *SD* = 1.90) completed an average of 1.50 fewer surveys (*SE* = 0.61) than present participants (*M* = 15.90, *SD* = 0.45; *t*(9.50) = −2.47, *p* < 0.05). There were a total of 16 mental health surveys sent to participants (i.e., four mental health outcome variables measured over four occasions) over the full study period. Again, Levene’s test indicated unequal variances between groups for this comparison (*F*(1, 28) = 74.14, *p* < 0.001), and the *t*-test for equal variances not assumed is reported.

Ten group comparisons were made in this series of independent-samples *t*-tests, and a Bonferroni correction [100] (i.e., *α* = 0.05/10) yielded a more conservative *p*-value of 0.005 as a threshold for statistical significance among these tests. Controlling for multiple comparisons with this value in mind, no statistically significant differences between those present or absent from post-testing were observed for adherence, additional fitness tracking, or mental health outcome variables at post-intervention. See Table S1 in Supplementary Materials for summaries of all tests performed here.

### 3.3. Intervention Adherence and Additional Fitness Tracking

On average, participants completed 29.86 workouts (*SD* = 6.38, range = 14.00–36.00) out of a possible 36 over the intervention period, or 82.94%. Two participants were excluded from this statistic as they only completed one (2.78%) and seven (19.44%) total workouts, respectively. Participants also completed an average of 43.03 RES practices (*SD* = 5.52, range = 31.00–48.00) out of a possible 48, or 89.65%. One participant was excluded for only having completed five (10.42%) total RES practices. Combining HIFT and RES practices, all participants completed an average of 72.10 intervention practices (*SD* = 11.16, range = 46.00–84.00) out of a possible 84, or 85.83%. The previous participant was also excluded from this statistic for only completing six (7.14%) of all intervention practices. Finally, participants completed an average of 15.40 outcome variable measurements (*SD* = 1.33, range = 12.00–16.00) out of a possible 16 (i.e., four outcome variable measurements over four measurement occasions), or 96.25%.

Regarding additional fitness tracking, participants reported an average of 3.25 additional workouts per week (*SD* = 1.90, range = 1.00–7.71) beyond those prescribed during the intervention. Two participants were excluded from this statistic for missing data and an outlying mean value of 12.38 additional workouts per week, respectively. Mean additional minutes of weekly exercise was also reported at 238.04 min (*SD* = 182.95, range = 60.00–760.00), or about an additional 4 h. This value dropped to 187.78 min (*SD* = 107.92, range = 60.00–480.00), or just over an additional 3 h per week, with three outliers removed (627.50, 633.33, and 760.00 additional min, respectively). Types of additional exercise varied widely among participants, but broad categories included various cardiorespiratory activities, resistance training, outdoor activities, mind/body exercises, and mixed martial arts (see Table S2 in Supplementary Materials for summaries of all reported exercise types). Participants’ mean intensity of additional weekly exercise was reported at 13.49 (*SD* = 2.07, range = 8.33–20.00) out of a possible 20 (i.e., maximal exertion), as rated on Borg’s 6–20 Rating of Perceived Exertion (RPE) scale [104]. With three outliers emitted (average workout RPEs of 8.33, 17.50, and 20.00, respectively), this value dropped only slightly to 13.29 (*SD* = 1.16, range = 11.16–15.79). Lastly, it should also be noted that tracking of participants’ usual fitness routines did not occur in the observation period between study weeks 0 and 4, and a baseline for usual exercise was not established prior to the start of the intervention period.

### 3.4. Mental Health Outcome Mean Values

Mean values for the study’s four outcome variables (i.e., depressive symptoms, PTSSs, psychological resilience, and mental wellbeing) at each measurement occasion are presented below in Table 3. Outliers have not been removed from this table in order to fully characterize the distribution of participants’ scores. Changes over time among these variables will be described in the coming section.

### 3.5. Repeated-Measures T-Tests and Effect Sizes

Repeated-measures *t*-test results are presented in Table 4 (mental health outcomes between baseline and pre-intervention), Table 5 (anthropometrics and job-task performance variables between pre- and post-intervention), and Table 6 (mental health outcomes between pre- and post-intervention) below. Effect sizes are presented above as Hedges’ *g* values. This measure of effect size was chosen due to its correction factor that accounts for upward-biased estimates (i.e., as observed with Cohen’s *d*) [105], especially in smaller sample sizes [93,106].

Overall, no significant mean differences were observed for any mental health outcome variable between baseline and pre-intervention measurements (*p*’s > 0.05). This finding also stands when comparing mean differences against a Bonferroni corrected *p*-value of 0.013 for the four comparisons above. No anthropometrics or physical performance variables are presented above as these measurements had not taken place yet.

Significant mean differences between pre- (*M* = 22.91, *SD* = 3.40) and post-intervention (*M* = 25.09, *SD* = 3.94) were observed for number of stations completed during the T-CAC workout. Specifically, participants completed an additional 2.18 stations (*SD* = 1.51; *t*(19) = 6.46, *p* < 0.001) on average during the post-intervention T-CAC workout. This mean difference also remains statistically significant when controlling for multiple comparisons using a Bonferroni-corrected *p*-value of 0.01. Correspondingly, Hedges’ *g* effect size was also statistically significant for additional T-CAC stations completed. The effect size for change in T-CAC stations completed between pre- and post-intervention can be interpreted as a large effect, and mean difference of 1.42 *SD.* No other significant mean differences were observed for anthropometric or job-task performance outcomes between study weeks 4 and 17 (*p*’s > 0.05).

Significant mean differences between pre- and post-intervention were also observed for PTSS severity and psychological resilience. PCL-C scores decreased by 5.00 points on average between pre- (*M* = 33.33, *SD* = 14.46) and post-intervention measurements (*M* = 28.33, *SD* = 13.47; *t*(26) = −3.51, *p* = 0.002), matching the minimum threshold for reliable change in symptoms and treatment response (i.e., a change of 5.00 points) [107]. Hedges’ *g* effect sizes were also statistically significant for the reduction in PCL-C scores at post-intervention and can be interpreted as a medium-to-large effect. Finally, participants’ CD-RISC10 scores increased by an average of 2.19 points between pre- and post-intervention (*t*(26) = 2.51, *p* = 0.02) from 29.85 (*SD* = 5.70) to 32.04 (*SD* = 5.73). The corresponding Hedges’ *g* effect size was 0.47 (95% CI [0.08, 0.85]), which falls just short of the medium-sized effect threshold of 0.50. Only the mean difference in PCL-C scores remains statistically significant when the Bonferroni-corrected *p*-value of 0.013 is utilized. No other significant mean differences were observed for anthropometric or outcome variables between study weeks 4 and 17 (*p*’s > 0.05).

### 3.6. Multilevel Modeling Results

Intraclass correlation coefficients (ICCs) were first calculated to observe the partitioning of variance across the four mental health outcomes at model levels 1 and 2. The ICC for depressive symptoms was calculated at 0.70, indicating that 70% of total outcome variance over time can be attributed to between-person constant mean differences of the random intercept at level 2 (i.e., differences in symptoms between participants), while 30% is due to remaining within-person variation around participants’ mean values (i.e., within-person variance of residuals) at level 1 [103]. Correspondingly, the ICCs for PTSSs, psychological resilience, and mental wellbeing were 0.84, 0.73, and 0.74, respectively. Therefore, the majority of outcome variation for all four mental health outcomes can be ascribed to cross-sectional differences between persons at level 2, rather than change over time within persons at each measurement occasion [103].

#### 3.6.1. Main Effects of Demographics on Mental Health Outcomes

Main effects were estimated for demographic variables and mean-centered baseline scores for all mental health outcomes centered at the pre-intervention measurement (week 4) and are presented in Table S3 (depressive symptoms), S4 (PTSSs), S5 (psychological resilience), and S6 (mental wellbeing) in Supplementary Materials. Subsequent conditional models retained mean-centered baseline scores as covariates due to statistically significant Wald tests for each fixed effect, namely PHQ-9 (*F*(1, 28) = 38.78, *p* < 0.001), PCL-C (*F*(1, 26) = 165.84, *p* < 0.001), CD-RISC10 (*F*(1, 27) = 56.38, *p* < 0.001), and WEMWBS (*F*(1, 26) = 47.30, *p* < 0.001) baseline scores.

For depressive symptom severity, participants’ grand mean PHQ-9 score at pre-intervention was 3.12 (*SE* = 0.58, *t* = 5.39, *p* < 0.001) as indicated by the unconditional model for the means (Model 1), with scores corresponding with “minimal” depression [108]. After accounting for growth, the unconditional growth model (Model 2) reported a mean PHQ-9 score (i.e., intercept) of 3.63 (*SE* = 0.63, *t* = 5.74, *p* < 0.001) at the start of the intervention and an average decline of 0.08 points per week over the intervention period (*SE* = 0.04, *t* = −2.02, *p* = 0.048). The conditional growth model (Model 3) indicated a significant positive fixed effect of mean-centered baseline PHQ-9 score (*B* = 0.76, *SE* = 0.12, *t* = 6.28, *p* < 0.001) that was retained in all successive models. This positive fixed effect can be interpreted as participants with higher baseline PHQ-9 scores also displaying higher scores at pre-intervention, on average. However, growth (i.e., change over time) was not statistically significant in Models 4–7 and 9 (*p*’s > 0.05). Predictors in these models for Age_MC_ (*p* = 0.40), education (*p* = 0.94), rank (*p* = 0.10), years of service_MC_ (*p* = 0.18), and relationship status (*p* = 0.72; i.e., Models 4–7 and 9, respectively) also did not significantly affect participants’ PHQ-9 scores. In Models 8 and 10, weekly decline in PHQ-9 score was statistically significant when controlling for participants’ race/ethnicity (*B* = −0.07, *SE* = 0.04, *t* = −2.01, *p* = 0.049) and sex (*B* = −0.07, *SE* = 0.04, *t* = −2.01, *p* = 0.049), respectively, although the fixed effects of race/ethnicity (*F*(1, 27) = 0.08, *p* = 0.78) and sex (*F*(1, 27) = 0.39, *p* = 0.54) were not statistically significant in and of themselves. In summary, depressive symptoms over the intervention period were unaffected by participants’ demographics, and change in PHQ-9 scores was not statistically significant overall (*p*’s > 0.05).

Concerning PTSS severity, participants’ grand mean PCL-C score at pre-intervention was 29.76 (*SE* = 2.36, *t* = 12.60, *p* < 0.001) according to the unconditional model for the means (Model 1). For reference, a PCL-C score of 44 relates to the highest diagnostic efficiency for probable PTSD diagnosis [76]. Significant weekly change over time in PCL-C score (*B* = −0.35, *SE* = 0.10, *t* = −3.61, *p* < 0.001) was also indicated by the unconditional growth model (Model 2), as well as a mean score of 32.12 (*SE* = 2.46, *t* = 13.08, *p* < 0.001) at pre-intervention. That is, on average, PCL-C scores decreased by 0.35 points per week over the intervention period. Significant change in PCL-C score over time (*B* = −0.34, *SE* = 0.10, *t* = −3.26, *p* = 0.002) was also observed when accounting for baseline PCL-C scores (*B* = 0.94, *SE* = 0.07, *t* = 13.15, *p* < 0.001) in Model 3, as well as all other models. This positive fixed effect also signifies that higher PCL-C scores at baseline are related to higher scores at pre-intervention, on average. While no other demographic fixed effects were statistically significant by themselves (Age_MC_ [*p* = 0.87], education [*p* = 0.75], rank [*p* = 0.69], years of service_MC_ [*p* = 0.80], race/ethnicity [*p* = 0.62], relationship status [*p* = 0.71], and sex [*p* = 0.11] in Models 4–10, respectively), growth remained significant (*p* < 0.01) across all models for PTSS severity centered at pre-intervention. Therefore, participants’ demographics had no effect on PTSS severity over the intervention period, but PCL-C scores were shown to decrease modestly week by week (*p*’s < 0.01).

Regarding psychological resilience, participants’ grand mean CD-RISC10 score for psychological resilience at pre-intervention was 31.18 (*SE* = 0.90, *t* = 34.53, *p* < 0.001) according to Model 1. Model 2 also indicated significant change in participants’ psychological resilience over time (*B* = 0.14, *SE* = 0.05, *t* = 2.74, *p* = 0.008), with a mean score of 30.20 (*SE* = 0.97, *t* = 31.08, *p* < 0.001) at pre-intervention. In other words, CD-RISC10 scores increased by an average of 0.14 points per week during the intervention period. Growth also remained statistically significant (*B* = 0.14, *SE* = 0.05, *t* = 2.63, *p* = 0.01) with the addition of baseline CD-RISC10 scores (*B* = 0.70, *SE* = 0.09, *t* = 7.64, *p* < 0.001) as a covariate in Model 3. Again, it can be said that higher CD-RISC10 scores at baseline corresponded with higher scores at pre-intervention, on average, as well. Congruent with models for PTSSs, no other demographic fixed effects were statistically significant (Age_MC_ [*p* = 0.91], education [*p* = 0.51], rank [*p* = 0.55], years of service_MC_ [*p* = 0.54], race/ethnicity [*p* = 0.85], relationship status [*p* = 0.13], and sex [*p* = 0.69]), but growth remained significant (*p* < 0.05) for all remaining models. That is, demographics had no effect on participants’ psychological resilience over the intervention period, but CD-RISC10 scores were shown to increase slightly week by week (*p*’s < 0.05).

Lastly, for mental wellbeing, participants’ grand mean WEMWBS score at pre-intervention was 51.24 (*SE* = 1.48, *t* = 34.66, *p* < 0.001) according to Model 1. The unconditional growth model (Model 2) indicated no significant change in WEMWBS score over time (*B* = 0.16, *SE* = 0.09, *t* = 1.77, *p* = 0.08) and reported a mean WEMWBS score of 50.19 (*SE* = 1.59, *t* = 31.54, *p* < 0.001) at pre-intervention. However, the addition of mean-centered baseline WEMWBS scores (*B* = 0.83, *SE* = 0.12, *t* = 6.88, *p* < 0.001) as a covariate in Model 3 did result in a statistically significant fixed effect for growth (*B* = 0.21, *SE* = 0.09, *t* = 2.40, *p* = 0.02). Growth remained statistically significant (*p* < 0.05) in Models 4–10 for mental wellbeing at pre-intervention, even though previous *t*-test results indicated no significant mean difference in WEMWBS scores between pre- and post-intervention (*t*(25) = 1.71, *p* > 0.05). The positive fixed effect of baseline scores also signifies the relationship between baseline and pre-intervention scores, in that higher scores at baseline are related to higher scores at pre-intervention as well, on average.

Main effects for participants’ fire department rank (*F*(1, 25) = 6.24, *p* = 0.02) and centered years of service (*F*(1, 25) = 5.30, *p* = 0.03) were significant predictors of mental wellbeing at pre-intervention. For rank, those ranked either Captain or Battalion Chief (53% of the sample) with average mental wellbeing at baseline saw a small increase in WEMWBS scores of 0.21 points per week (*SE* = 0.09, *t* = 7.11, *p* < 0.001) during the intervention period. However, increasing rank from Firefighter or Engineer to Captain or Battalion Chief was related to lower mental wellbeing overall (*B* = −4.32, *SE* = 1.73, *t* = −2.50, *p* = 0.02). This mean difference exceeds the minimum value of three points for clinical change (i.e., improvement or decline) for mental wellbeing as measured by the WEMWBS [88]. Similar results were observed for mean-centered years of service, in that an increase in years employed in the fire service was related to slightly decreased mental wellbeing as well (*B* = −0.25, *SE* = 0.11, *t* = −2.30, *p* = 0.03). However, growth remained statistically significant when accounting for participants’ rank (*B* = 0.21, *SE* = 0.09, *t* = 2.44, *p* = 0.02) and years of service (*B* = 0.21, *SE* = 0.09, *t* = 2.40, *p* = 0.02). There were no other significant demographic fixed effects on mental wellbeing (Age_MC_ [*p* = 0.51], education [*p* = 0.86], race/ethnicity [*p* = 0.89], relationship status [*p* = 0.43], and sex [*p* = 0.69]).

#### 3.6.2. Main Effects of Intervention Adherence and Additional Fitness Tracking on Anthropometrics and Job-Task Performance

Next, effects of intervention adherence and additional fitness tracking were assessed on anthropometrics (i.e., bodyweight [BW], lean body mass [LBM], body mass index [BMI], and percent body fat [%BF]) and job-task performance (i.e., Time-Critical Athlete Challenge [T-CAC] stations completed). Unlike previous MLMs, all conditional models excluded mean-centered pre-intervention values as covariates. Results are presented in Tables S7 (BW), S8 (LBM), S9 (BMI), S10 (%BF), and S11 (T-CAC) in Supplementary Materials.

Concerning BW (kg), the unconditional mean model (Model 1) indicated participants’ grand mean value was 90.87 kg (*SE* = 2.22, *t* = 40.94, *p* < 0.001) at pre-intervention. The addition of growth in Model 2 was not statistically significant (*B* = −0.05, *SE* = 0.04, *t* = −1.48, *p* = 0.16), indicating no change over time for participants’ BW. Growth remained nonsignificant (*p*‘s > 0.05) in all remaining models (3–11), as well as all other predictors for intervention adherence and additional fitness tracking (*p*’s > 0.05). These results echo previously mentioned repeated-measures *t*-test results (*t*(19) = 1.50, *p* > 0.05), indicating no significant change over time in participants’ BW between pre- and post-intervention.

For LBM (kg), Model 1 reported a grand mean value of 71.85 kg (*SE* = 1.69, *t* = 42.45, *p* < 0.001) at pre-intervention. The unconditional growth model (Model 2) indicated no significant change in LBM over time (*B* = −0.03, *SE* = 0.04, *t* = −0.96, *p* = 0.35), echoing the corresponding repeated-measures *t*-test result (*t*(19) = 1.06, *p* > 0.05) for this variable. No significant change over time in participants’ LBM was observed between pre- and post-intervention. However, significant predictors were observed in Models 5, 8, 10, and 11. In Model 5, combined adherence_STD_ (*F*(1, 24) = 6.42, *p* = 0.02) and the combined adherence_STD_ × growth interaction (*F*(1, 18) = 4.43, *p* = 0.049) significantly predicted LBM at pre-intervention when controlling for mean-centered additional workouts, additional minutes of exercise, and RPE of additional exercise per week. That is, those who displayed increased combined HIFT and RES adherence over the intervention period tended to have more LBM at pre-intervention on average (*B* = 6.32, *SE* = 2.49, *t* = 2.53, *p* = 0.02), but this nonsignificant change in LBM (growth: *F*(1, 18) = 0.13, *p* = 0.72) associated with increased combined adherence_STD_ decreased, on average, over time (*B* = −0.13, *SE* = 0.06, *t* = −2.10, *p* = 0.049).

In Model 8, HIFT adherence_STD_ significantly predicted LBM at pre-intervention (*F*(1, 24) = 5.03, *p* = 0.03) when controlling for additional fitness tracking, in that those with increased HIFT adherence over the intervention period tended to also have more LBM at pre-intervention on average (*B* = 4.52, *SE* = 2.02, *t* = 2.24, *p* = 0.03). Lastly, the RES adherenceSTD × growth interaction was significant in Models 10 (*F*(1, 18) = 5.37, *p* = 0.03) and 11 (*F*(1, 18, 5.71, *p* = 0.03). However, both RES adherence_STD_ (Model 10: *F*(1, 28) = 1.33, *p* = 0.26; Model 11: *F*(1, 24) = 3.27, *p* = 0.08) and growth (Model 10: *F*(1, 18) = 0.00, *p* = 0.98; Model 11: *F*(1, 18) = 0.00, *p* = 0.96) were not statistically significant in either model. No other predictors were statistically significant (*p*’s > 0.05) in any other model for LBM.

Regarding participants’ BMI (kg·m^−2^), Model 1 indicated a grand mean value of 28.16 (*SE* = 0.52, *t* = 54.16, *p* < 0.001) at pre-intervention. Growth was not statistically significant in Model 2 (*B* = −0.02, *SE* = 0.01, *t* = −1.54, *p* = 0.14), reiterating the associated repeated-measures *t*-test result (*t*(19) = −1.51, *p* > 0.05). No significant change was observed in BMI between pre- and post-intervention. Further, no other predictors produced significant effects on BMI (*p*’s > 0.05).

For %BF, Model 1 relayed a sample grand mean of 20.74% (*SE* = 0.94, *t* = 22.01, *p* < 0.001) at pre-intervention. The addition of growth in Model 2 was also not statistically significant (*B* = −0.004, *SE* = 0.03, *t* = −0.17, *p* = 0.87), indicating no change in %BF over the intervention period. This result paralleled the associated repeated-measures *t*-test for %BF (*t*(19) = −0.05, *p* > 0.05) as well. Like BMI, no other predictors produced significant effects on participants’ %BF over the intervention period (*p*’s > 0.05).

Finally, for T-CAC stations completed, participants’ grand mean value at pre-intervention was 22.93 (*SE* = 0.73, *t* = 31.24, *p* < 0.001) according to Model 1. For reference, the T-CAC was used as the simulated job-task performance test in this study. The T-CAC consisted of eight stations and lasted 20 min, wherein participants completed as many stations as possible before the time limit elapsed. Growth was statistically significant in Model 2 (*F*(1, 19) = 49.71, *p* < 0.001), indicating an average increase of 0.18 stations completed (*SE* = 0.03, *t* = 7.05, *p* < 0.001) per week over the intervention period. The proprietary nature of this test makes it difficult to assess the “real-world” significance of this difference, but participants performed the T-CAC with maximal effort and treated it as a graded CPAT-like test. Growth remained statistically significant in all other models (*p*’s < 0.001), indicating significant change over time in parallel with the associated repeated-measures *t*-test (*t*(19) = 6.46, *p* < 0.001). Additionally, significant main effects were observed for combined adherence_STD_ in Models 3 and 4, as well as for HIFT adherence_STD_ in Models 6–8.

Combined adherence_STD_ significantly predicted T-CAC performance at pre-intervention in Model 3 (*F*(1, 28) = 7.25, *p* = 0.01), indicating that those with increased HIFT and RES adherence over the intervention period tended to also complete more T-CAC stations at pre-intervention, on average (*B* = 1.75, *SE* = 0.65, *t* = 2.69, *p* = 0.01). The same can be said of combined adherence_STD_ (*F*(1, 28) = 7.65, *p* = 0.01; *B* = 1.80, *SE* = 0.65, *t* = 2.77, *p* = 0.01) when controlling for the nonsignificant combined adherence_STD_ × growth interaction (*p* = 0.44) in Model 4. Similarly, HIFT adherence_STD_ significantly predicted participants’ T-CAC performance at pre-intervention, in that those with increased HIFT adherence over the intervention period also tended to complete more stations on the pre-intervention T-CAC test, on average. This was observed in Model 6 (*F*(1, 28) = 8.76, *p* = 0.006; *B* = 1.89, *SE* = 0.64, *t* = 2.96, *p* = 0.006); in Model 7 (*F*(1, 28) = 9.50, *p* = 0.005; *B* = 1.96, *SE* = 0.64, *t* = 3.08, *p* = 0.005) when controlling for the nonsignificant HIFT adherence_STD_ × growth interaction (*p* = 19); and in Model 8 (*F*(1, 24) = 5.25, *p* = 0.03; *B* = 1.81, *SE* = 0.79, *t* = 2.29, *p* = 0.03) when controlling for the nonsignificant HIFT adherence_STD_ × growth interaction (*p* = 18), as well as mean-centered additional workouts (*p* = 0.77), additional minutes of exercise (*p* = 0.20), and RPE of additional exercise (*p* = 0.76) per week over the intervention period. No other predictors were statistically significant in any other model (*p*’s > 0.05) for T-CAC performance.

#### 3.6.3. Main Effects of Intervention Adherence and Additional Fitness Tracking on Mental Health Outcomes

Finally, effects of intervention adherence and additional fitness tracking were assessed on mental health outcomes centered at the pre-intervention measurement (week 4) and are presented in Tables S12 (depressive symptoms), S13 (PTSSs), S14 (psychological resilience), and S15 (mental wellbeing) in Supplementary Materials. All conditional models retained mean-centered baseline scores as covariates due to statistically significant Wald tests for each fixed effect, namely mean-centered baseline PHQ-9 scores (*F*(1, 28) = 38.78, *p* < 0.001), PCL-C scores (*F*(1, 26) = 165.84, *p* < 0.001), CD-RISC10 scores (*F*(1, 27) = 59.45, *p* < 0.001), and WEMWBS scores (*F*(1, 26) = 47.30, *p* < 0.001).

For depressive symptoms, participants’ grand mean PHQ-9 score at pre-intervention was 3.12 (*SE* = 0.58, *t* = 5.39, *p* < 0.001) as indicated by Model 1. Similar to previous models, this value corresponds with “minimal” depression according to the PHQ-9 [108]. With the inclusion of growth in Model 2, participants’ mean PHQ-9 score at pre-intervention increased to 3.62 (*SE* = 0.63, *t* = 5.70, *p* < 0.001). Growth was not statistically significant in this model, however (*p* = 0.07). Model 3 indicated a significant positive fixed effect of mean-centered baseline PHQ-9 score (*F*(1, 28) = 38.78, *p* < 0.001; *B* = 0.76, *SE* = 0.12, *t* = 6.22, *p* < 0.001) that was retained in all successive models. Growth was not statistically significant in Models 3–11, though (*p*’s > 0.05). Of these 12 models, the lone significant predictor was HIFT adherence_STD_ (*F*(1, 27) = 6.48, *p* = 0.02) in Model 7, which controlled for the main effects of baseline PHQ-9 score (*B* = 0.77, *SE* = 0.11, *t* = 6.97, *p* < 0.001) and HIFT adherence_STD_ (*B* = −0.92, *SE* = 0.36, *t* = −2.55, *p* = 0.02). The intercept in Model 7 was also statistically significant (*Y* = 3.63, *SE* = 0.43, *t* = 8.44, *p* < 0.001), but growth was not (*p* = 0.07).

The significant predictor weight for HIFT adherence_STD_ indicates that those who displayed increased adherence to HIFT workouts over the intervention period tended to report lower PHQ-9 scores at pre-intervention, when also controlling for baseline scores. Lastly, growth was significant (*F*(1, 52) = 5.15, *p* = 0.03) in Model 12, which controlled for baseline score (*F*(1, 23) = 43.07, *p* < 0.001), RES adherence_STD_ (*p* = 0.67), the RES adherence_STD_ × growth interaction (*p* = 0.21), additional workouts_MC_ (*p* = 0.12), additional minutes of exercise_MC_ (*p* = 0.83), and RPE of additional workouts_MC_ (*p* = 0.48). The intercept for this model was also significant at 3.71 (*SE* = 0.47, *t* = 7.97, *p* < 0.001). That is, PHQ-9 scores decreased by an average of 0.09 points (*SE* = 0.77, *t* = −2.27, *p* = 0.03) per week throughout the intervention period when controlling for the aforementioned covariates in Model 12. Overall, no significant change was observed in depressive symptoms over the intervention period, and PHQ-9 scores were only affected by HIFT adherence.

Next, Model 1 concerning PTSSs indicated a grand mean PCL-C value of 29.76 at pre-intervention (*SE* = 2.36, *t* = 12.60, *p* < 0.001). The addition of growth in Model 2 was also significant (*F*(1, 54) = 11.13, *p* = 0.002), indicating an average decline in PCL-C scores of 0.32 points (*SE* = 0.10, *t* = −3.34, *p* = 0.002) per week over the intervention period. Baseline PCL-C scores were also a significant predictor (*F*(1, 26) = 165.84, *p* < 0.001) in Model 3, alongside growth (*F*(1, 50) = 9.45, *p* = 0.003). The mean decline in PCL-C scores per week remained at 0.32 points (*SE* = 0.10, *t* = −3.07, *p* = 0.003) in Model 3, and higher baseline scores coincided with higher scores at pre-intervention, on average. In all remaining models (4–11), only the model intercepts, mean-centered baseline scores, and growth remained statistically significant at *p* < 0.05. There were no significant fixed effects (*p*’s > 0.05) for any intervention adherence components (combined adherence_STD_, HIFT adherence_STD_, and RES adherence_STD_), their interactions with growth, or additional fitness tracking variables (additional workouts_MC_, additional minutes of exercise_MC_, and RPE of additional workouts_MC_) on PTSSs when centered at pre-intervention. The models suggest that, while participants’ PTSS severity declined week by week on average over the intervention period, adherence to HIFT workouts and RES practices had little to no effect on symptoms overall.

For psychological resilience, Model 1 indicated a grand mean CD-RISC10 score of 31.18 (*SE* = 0.90, *t* = 34.53, *p* < 0.001) at pre-intervention. The addition of growth in Model 2 was statistically significant (*F*(1, 54) = 6.01, *p* = 0.02), indicating that CD-RISC10 scores increased an average of 0.13 points (*SE* = 0.05, *t* = 2.45, *p* = 0.02) each week over the intervention period. Growth remained statistically significant (*p* = 0.02) in Model 3, which controlled for participants’ baseline CD-RISC10 scores (*F*(1, 27) = 56.38, *p* < 0.001). Higher baseline scores were related to higher scores at pre-intervention as well, according to Model 3 (*B* = 0.70, *SE* = 0.09, *t* = 7.51, *p* < 0.001). Growth (*p*’s < 0.05) and baseline scores (*p*’s < 0.001) remained the only statistically significant predictors in all other models for psychological resilience, implying significant change over time but little influence of intervention adherence or additional fitness tracking variables on this outcome.

Finally, for mental wellbeing, Model 1 indicated a grand mean WEMWBS value of 51.24 at pre-intervention (*SE* = 1.48, *t* = 34.66, *p* < 0.001). The addition of growth in Model 2 was not significant (*F*(1, 54) = 3.13, *p* = 0.08). Baseline WEMWBS scores were a significant predictor (*F*(1, 26) = 47.30, *p* < 0.001; *B* = 0.83, *SE* = 0.12, *t* = 6.88, *p* < 0.001) in Model 3, as well as growth (*F*(1, 50) = 5.77, *p* = 0.02). According to this model, participants’ WEMWBS scores increased an average of 0.21 points (*SE* = 0.09, *t* = 2.40, *p* = 0.02) over the intervention period, and higher baseline scores coincided with higher scores at pre-intervention, on average, as well. Growth remained statistically significant (*p*’s < 0.05) in remaining Models 4–12 when controlling for significant baseline scores (*p*’s < 0.001), but no significant fixed effects were observed for intervention components, their interactions with growth, or additional fitness tracking variables on participants’ mental wellbeing (*p*’s > 0.05). Given the lack of omnibus change in Model 2 and the associated WEMWBS repeated-measures *t*-test result (*t*(25) = 1.71, *p* > 0.05), change over time in mental wellbeing from pre- to post-intervention was not observed in this sample.

### 3.7. Mental Health Outcome Measurement Distributions

**Figure 2:**
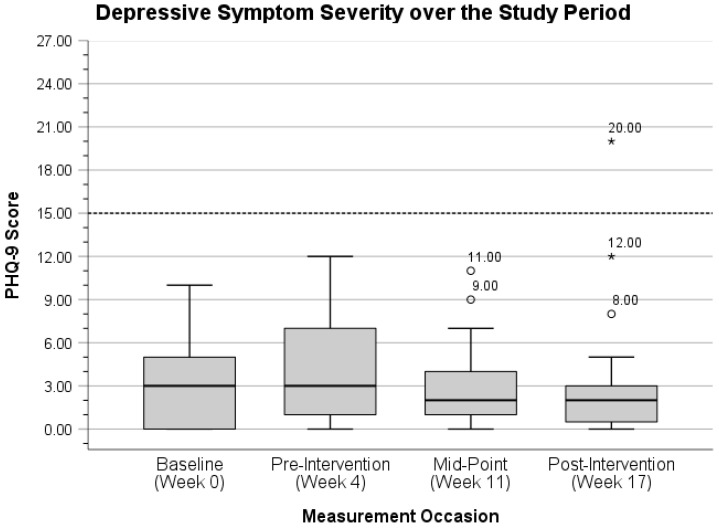
Boxplot of depressive symptoms (i.e., PHQ-9 scores) over the study period. Scores can range from 0 to 27. A cutoff score of 15 (dashed line) represents a likely threshold of major depression [75] (p. 611) and is marked above. Circles above represent mild outliers with values at least 1.5× the interquartile range below the first quartile or above the third quartile. Stars above represent extreme outliers with values at least 3.0× the interquartile range below the first quartile or above the third quartile.

Boxplots for each mental health variable were plotted to better visualize possible changes in outcome measurement distributions over the study period. Reference values for each variable are also provided below to better characterize participants’ symptom severity and mental health ratings over time. Distribution of depressive symptoms can be viewed in Figure 2 below.

**Figure 3:**
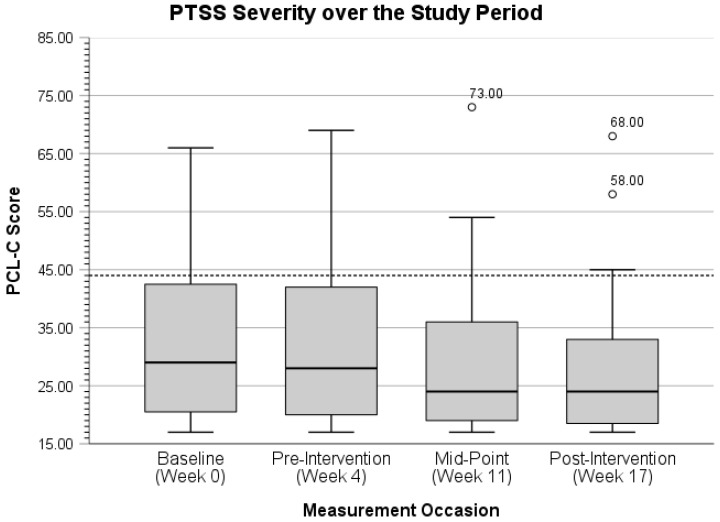
Boxplot of post-traumatic stress symptom severity (i.e., PCL-C scores) over the study period. Scores can range from 17 to 85. A score of 44 (dashed line) is a likely threshold of diagnosable PTSD [76] (p. 672) and is marked above. Circles above represent mild outliers with values at least 1.5× the interquartile range below the first quartile or above the third quartile.

With outliers removed (*n* = 2), participants’ mean PHQ-9 score at the midpoint measurement occasion decreased from 2.83 (*SD* = 2.71, range = 0.00–11.00) to 2.30 (*SD* = 1.90, range = 0.00–7.00). Similarly, participants’ mean PHQ-9 score at the post-intervention measurement occasion decreased from 2.93 (*SD* = 4.32, range = 0.00–20.00) to 1.63 (*SD* = 1.41, range = 0.00–5.00) with outliers removed (*n* = 3). Next, the distribution of PTSS severity over the study period is displayed in Figure 3 below.

**Figure 4:**
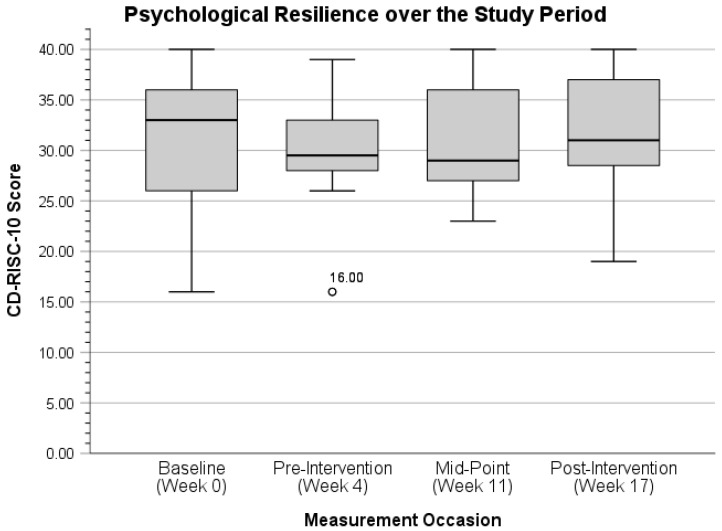
Boxplot of psychological resilience (i.e., CD-RISC10 scores) over the study period. Scores can range from 0 to 40. Circles above represent mild outliers with values at least 1.5× the interquartile range below the first quartile or above the third quartile.

With the single outlier removed from the midpoint measurement occasion, participants’ mean PCL-C score decreased from 31.00 (*SD* = 5.25, range = 17.00–23.00) to 27.75 (*SD* = 11.24, range = 17.00–54.00). Further, participants’ mean PCL-C score at post-intervention with outliers removed (*n* = 2) decreased from 28.33 (*SD* = 13.47, range 17.00–68.00) to 25.56 (*SD* = 9.30, range = 17.00–45.00). The distribution of psychological resilience over the study period is portrayed next in Figure 4 below.

**Figure 5:**
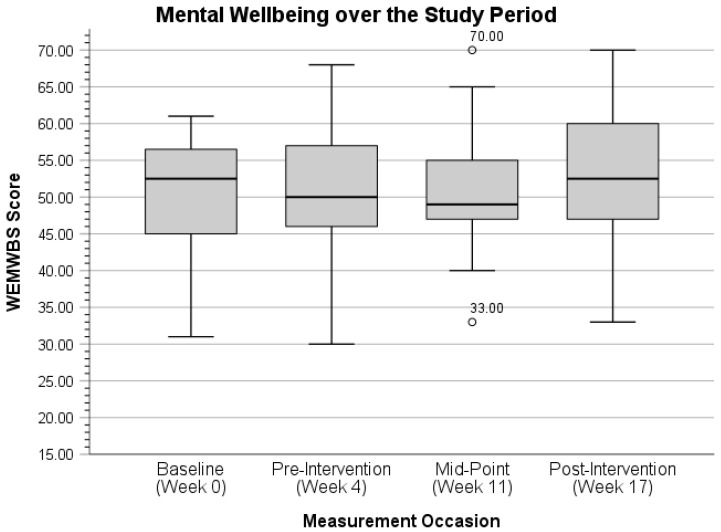
Boxplot of mental wellbeing (i.e., WEMWBS scores) over the study period. Scores can range from 14 to 70. Circles above represent mild outliers with values at least 1.5× the interquartile range below the first quartile or above the third quartile.

With two outliers (both scores of 16.00) removed at the pre-intervention measurement occasion, participants’ mean CD-RISC10 score increased slightly from 30.27 (*SD* = 5.60, range = 16.00–39.00) to 31.29 (*SD =* 4.19, range = 26.00–39.00). Lastly, the distribution of mental wellbeing over the study period is presented last in Figure 5 below.

With outliers removed (*n* = 2), participants’ mean WEMWBS score at midpoint decreased slightly from 50.48 (*SD* = 7.61, range = 33.00–77.00) to 50.41 (*SD* = 5.99, range = 40.00–65.00).

### 3.8. Post Hoc Sample Size and Power Analyses Results

For detecting change in depressive symptoms between pre- and post-intervention, given a sample size of 27 pairs (i.e., the number of participants that provided data at both occasions), two-tailed *α* = 0.05, and Hedges’ *g* effect size of −0.32, achieved power was 0.36, far from the standard of 0.80. The minimum sample size required to detect this effect was 79 pairs for a power of 0.80. Next, for detecting change in PTSSs between pre- and post-intervention (*n* = 27, two-tailed *α* = 0.05, Hedges’ *g* = −0.68), achieved power was 0.92. For an effect of this size, only 20 pairs were needed. Concerning changes in psychological resilience (*n* = 27, two-tailed *α* = 0.05, Hedges’ *g* = 0.48), achieved power fell short at 0.68. Here, G*Power indicated that a minimum sample size of 36 pairs was necessary to achieve a power of 0.80. Finally, for changes in participants’ mental wellbeing (*n* = 26, two-tailed *α* = 0.05, effect size = 0.34), achieved power also fell short at 0.38. The minimum sample size for a power of 0.80 was found to be 72 pairs to detect an effect of this size. Therefore, if judging purely by repeated-measures *t*-tests as indicators of omnibus change over the intervention period, at least 80 complete pairs of participant data would have been required to adequately identify significant mean differences in the current study.

## 4. Discussion

The purpose of this study was to investigate the impact of an occupationally-tailored, two-part intervention on mental health among career firefighters. The intervention combined both RES and HIFT to improve measures of mental health symptoms (i.e., depressive symptoms and PTSSs) and positive mental health (i.e., psychological resilience and mental wellbeing).

To better characterize the distribution of participants’ scores for depressive symptoms and PTSSs, one potential case (i.e., 3.33% of the sample) of major depression was observed at post-intervention with a PHQ-9 score of 20. Here, Spitzer et al. present a cutoff score of 15 as a likely threshold for major depression [75] (p. 611). For PTSS severity, Blanchard et al. regard a score of 44 as the value corresponding with the highest diagnostic efficiency for probable PTSD diagnosis [76] (p. 672). Seven participants (i.e., 23.33%) exceeded this threshold at baseline, six (i.e., 20%) exceeded this threshold at pre-intervention, five (i.e., 16.67%) met or exceeded this threshold at mid-intervention, and five (i.e., 16.67%) met or exceeded this threshold at post-intervention. Despite the sub-clinical focus of this study, all participants were provided with fire-department-specific mental health resources, as well as those at larger county, state, and national levels at all measurement occasions.

Regarding the significant increase in T-CAC stations completed at post-intervention, it could be postulated that the intervention was successful given the high percentage of adherence to HIFT workouts (over 80%). Another explanation could be that participants adjusted their exercise habits over the intervention period to improve their overall fitness and, ultimately, their post-intervention T-CAC performance. This result was implied in a previous study of career firefighters in which both experimental and control groups saw significantly improved performance on a modified CPAT when evaluated three times over a 14-week period [43]. Additionally, it is possible that participants improved T-CAC stations completed at post-intervention due to familiarization with the testing format as well, as was suggested by Hollerbach et al. [69] in a similar intervention. It is also possible that the requirement to undergo T-CAC post-testing also contributed to attrition at post-intervention. To this end, the lack of change in anthropometric measurements might also be attributed to the loss to follow-up of 10 participants, or 33.33% of the sample, at post-intervention.

Corthésy-Blondin et al. attribute attrition rates in excess of 30% to work schedule conflicts, deviation from standard procedures, and stigmas surrounding mental health among public safety personnel [109]. In addition, when designing behavioral health interventions for firefighters, Jahnke et al. [110] state that firefighters prefer interventions that interact with and are embedded in existing organizational contexts and cultures, rather than interventions that parallel or are separate from daily operations. Authors also elaborate that “the importation of specific interventions may disrupt the normal flow of these interactions” [110] (p. 122) and can ultimately be perceived as cumbersome by participants [111]. It is probable that aspects of the current intervention, despite their best intentions, could have been perceived in this way (e.g., 16 total mental health outcome measurements, pre- and post-intervention anthropometric and performance testing, seven total intervention protocols to complete weekly during the intervention, and weekly HIFT and RES ratings containing multiple additional questions). Even though coordination with Battalion Chiefs was frequently undertaken on all aspects of study implementation, and all HIFT workouts and RES practices were tailored to equipment availability and firefighting culture, the current study still lost 10 participants to follow-up. These participants were not polled as to their reasons why, and the feasibility of the current intervention, or others of similar design, could have benefited if this data was collected.

In terms of statistical validity, descriptive and graphical tests revealed departures from normality for almost all predictor and outcome variables. However, considering the small sample size (*N* = 30) and emphasis on ease of interpretation, no variables were transformed for MLMs apart from predictors that were mean-centered or standardized. Additionally, many demographic variables were collapsed down into dichotomous predictors in an attempt to better equalize group sizes and distributions of variance. Overall, these frequent departures from normality could have affected estimates of the random effects variances and covariances, and in turn, the fixed effects for all models [103]. Undoubtedly, a larger sample size with an accompanying increase in variability among the data could also assist in remedying these issues.

Further, while a definitive sample size required to detect significant effects in MLMs was not calculated in the current study, Tabachnick and Fidell do state that large sample sizes are needed even for models with only a few predictors (i.e., at least 60 participants when estimating five or fewer parameters [112]; p. 792). They also explain that statistical power in MLMs depends on compliance with model assumptions and grows with ICCs. While it is probable that a majority of MLMs in the current study violated underlying assumptions, ICCs were high and ranged between 0.70 and 0.84 for mental health outcome variables. Authors also state that power improves with more level 2 units (i.e., participants in the current study) and fewer level 1 units (i.e., measurement occasions) per level 2 unit [112]. The current study design accounted for this caveat, though unintentionally, concluding with four measurement occasions each among 30 participants. This design feature also met authors’ reference that 20 or more level 2 units were required to sufficiently test cross-level effects (i.e., interactions between level 1 and level 2 predictors, such as the combined adherence_STD_ [level 2, between-subjects predictor] × growth [level 1, within-subjects predictor] interaction in the current study) [112].

In comparison with other studies, the 17-week study period and 12-week intervention durations were chosen based on findings of previous research. Past intervention lengths reviewed by Rosenbaum et al. [38] included randomized controlled trials employing exercise (i.e., yoga, combined aerobic and RT, or stationary cycling) that spanned 6–12 weeks as a means to reduce symptoms of PTSD, with exercise groups showing significant reductions compared to controls [38]. Similar effects of exercise versus control groups have also been observed regarding depressive symptoms [38]. Further, a combined 12-week intervention of usual care (i.e., a combination of psychotherapy, pharmaceutical interventions, and group therapy) with RT and low-intensity aerobic exercise (AE) by Rosenbaum et al. [36] also resulted in significant reductions in both PTSD and depressive symptoms compared to a usual-care-only control group. Specific to HIFT, Sempf and Thienes [113] proposed an 8-week firefighter-specific program for improving physical fitness, health, and safety among this group. Ultimately, a 12-week intervention period was chosen to best actualize a combination of physical performance and mental health improvements among this study group.

Pertaining to increasing psychological resilience among public safety personnel, a meta-analysis of six studies revealed a Hedges’ *g* effect size of 0.60 (95% CI [0.34, 0.85]) for mindfulness-based interventions delivered electronically [114]. For comparison, the Hedges’ *g* value for changes in psychological resilience in the current study was 0.48 (95% CI [0.08. 0.87]). Further, a review by Rosenbaum et al. [38] presented a Hedges’ *g* effect size of −0.31 (95% CI [−0.60, −0.02]) when comparing the effects of exercise over controls at reducing PTSS severity. Further, another study by Rosenbaum et al. [36] reported a mean difference of −5.40 points (95% CI [−10.5, −0.3]) on the PCL-C between an exercise (i.e., RT and low-intensity AE) plus usual-care group and a usual-care-only control group. In the current study, PTSS severity was reduced by an average of 5.00 points (*SD* = 7.40, 95% CI [−7.93, −2.07]) between pre- and post-intervention as measured by the PCL-C. This equates to a Hedges’ *g* effect size of −0.67 (95% CI [−1.07, −0.25]) and expands the literature on the possible mental health effects of one such combined intervention employing HIFT and RES among this population.

Hollerbach et al. reported a 75% adherence rate to HIFT workouts [69], comparable to the current study’s. Also, loss to follow-up from an initial sample of 13 to 10 equated to an attrition rate of 23.08%, lower than the 33.33% observed in the current study. However, unlike the current study, no mental health outcomes were measured. While Hollerbach et al. [69] first utilized the TF20 platform among fire academy recruits, Day et al. [70] were some of the first to employ TF20 programming among volunteer firefighters. In turn, the current study extends the literature base by being one of the first to implement the TF20 platform among career firefighters. These two interventions embody the most comparable to the current study.

Concerted effort in firefighter research has investigated the promotion of psychological resilience to protect against PTSD and improve mental health overall [44,47,49,57,60,61,62,63,64,115,116,117,118,119,120,121,122]. Research detailing the effectiveness of exercise-based interventions in affecting indices of mental wellbeing and resilience are also beginning to emerge [51,123,124,125,126,127,128,129]. The current study results indicated that participants’ scores for PTSS severity decreased over the duration of the two-part intervention, while psychological resilience scores simultaneously increased.

Therefore, future research should continue to examine the utility of similar combined mental-physical approaches on improving mental health among firefighters, other public safety personnel, and the larger population as a whole. However, when establishing separate study groups in future firefighter interventions, researchers should be aware of the high likelihood of cross-talk between groups [43,110], as was the case in the current study; participants frequently worked through RESpractices (e.g., some participants reported discussing and journaling on weekly RES practices together) and HIFT workouts with each other, as well as other station-mates not enrolled in the study. While this tight cohesion and social support are vital for occupational performance and safety of firefighters in time-critical situations [130,131,132], this can prove challenging when designing experimental studies to better isolate treatment effects from potential confounders. Researchers should therefore consider matching participants across separate departments, when feasible, to help control for this interaction between participants.

## 5. Conclusions

This investigation was one of the first to formally employ TF20 R2R programming amongst a sample of career firefighters, expanding the knowledge base beyond fire academy recruits and volunteer counterparts. Second, the study utilized four mental health measurements to better characterize changes in participants’ mental health over time. Lastly, the current intervention also informs emerging research detailing the effectiveness of remotely delivered, web-based study protocols at improving mental health among firefighters, especially pertaining to the weekly frequency of email and text-message communications.

However, the current study is not without its limitations. The study was underpowered and impacts the generalizability of results to broader firefighting contexts. The self-reported nature of mental health questionnaires, intervention adherence, and additional fitness tracking could have also resulted in data inaccuracies. Future research should strongly emphasize the practicality and accuracy of data collection to ensure the validity of statistical modeling and results. Third, workout equipment availability varied widely across fire stations and departments in the current study. However, most participants reported that they improvised with available equipment or utilized local gyms to perform some workouts instead. Fourth, although completely intrinsic to this study population, participants’ 48 h on-duty/96 h off-duty shift schedules could have negatively impacted intervention fidelity as well, as participants frequently fluctuated between performing HIFT workouts and RES practices in-station or at home over the 12-week intervention period.

Due to the nature of their professions, public safety personnel require more engaging methods for the delivery of mental health information, resources for continuous career-long access to mental health knowledge, and more systematic approaches for improving workplace culture specific to mental health [111]. To this end, more effective RES programming might also entail enrollment into programs spanning many months, if not years, that can provide sustained access to mental health resources and culturally competent professionals whom participants can call upon when needed [110,111,132]

Access to a repository of psychological interventions, best practices, and culturally relevant applications might also assist firefighters in career-long access to knowledge. This would also better allow them the flexibility, autonomy, and ability to continually bolster and maintain their mental health on their own time and pace. While the financial and practical feasibility of such programs would indeed vary by department size, resource availability, and employment status (i.e., volunteer vs. career), future research should examine the utility of these extended resources among this group. Additionally, while the availability of mental health interventions is immense [111], definitive evidence of best practices remains scarce [109]. Future work should also focus on collaboration between relevant stakeholders when designing and implementing mental health programs to expand uptake and improve outcomes among participants [123].

## Figures and Tables

**Table 1:** Demographics and other characteristics of study participants (*n* = 30).

Characteristic		*n*	%
Sex	Female	3	10.00
	Male	27	90.00
Rank	Firefighter	8	26.66
	Engineer	6	20.00
	Captain	10	33.33
	Battalion Chief	6	20.00
Relationship Status	Single	3	10.00
	In a relationship	2	6.66
	Married or domestic partnership	24	80.00
	Divorced or separated	1	3.33
Education Level	Some college but no degree	2	6.66
	Associate degree	13	43.33
	Bachelor degree	13	43.33
	Graduate degree	2	6.66
Race	White	27	90.00
	Other	1	3.33
	Don’t know	1	3.33
	Prefer not to say	1	3.33
Ethnicity	Not Hispanic	26	86.66
	Hispanic	3	10.00
	Prefer not to say	1	3.33
**Characteristic**		***M*** **(*SD*)**	**Range**
Age (years)		39.70 (7.71)	23.00–57.00
Years in fire service		15.43 (8.37)	1.00–35.00

**Table 2:** Participants’ anthropometric and job-task performance measurements.

	Pre-Intervention, Full Sample (*n* = 30)		Pre-Intervention, Returning Sample (*n* = 20)		Post-Intervention, Returning Sample (*n* = 20)	
Variable	*M* (*SD*)	Range	*M* (*SD*)	Range	*M* (*SD*)	Range
Height (cm)	179.41 (7.68)	160.02–190.50	181.36 (6.61)	170.18–190.50	181.36 (6.61)	170.18–190.50
Weight (kg)	91.09 (12.65)	64.33–120.95	93.33 (10.33)	77.27–120.95	92.61 (9.61)	78.41–115.77
LBM (kg)	71.99 (9.51)	50.44–86.67	74.83 (7.23)	63.15–86.67	74.34 (7.34)	63.24–87.40
BMI (kg·m^−2^)	28.23 (2.96)	22.48–35.25	28.36 (2.49)	22.48–33.33	28.15 (2.32)	22.81–31.90
%BF	20.76 (5.44)	11.10–31.80	19.57 (5.26)	11.10–31.80	19.56 (4.76)	11.30–29.10
T-CAC (stations)	22.12 (3.83)	15.00–30.00	22.91 (3.40)	16.00–29.33	25.09 (3.94)	17.00–32.47

BMI, body mass index; cm, centimeters; LBM, lean body mass; kg, kilograms; kg·m-2, kilograms per meter squared; T-CAC, Time-Critical Athlete Challenge; %BF, body fat percentage. T-CAC (stations) reflects the total number of stations participants completed during the 20 min, 8-station circuit workout.

**Table 3:** Mean values for outcome variables at each measurement occasion.

Occasion	PHQ-9			PCL-C		
	*M* (*SD*)	Range	Missing (%)	*M* (*SD*)	Range	Missing (%)
Baseline (Week 0)	3.43 (3.22)	0–10	0 (0.00)	32.11 (13.20)	17–66	2 (6.66)
Pre-Intervention (Week 4)	3.77 (3.61)	0–12	0 (0.00)	32.33 (14.28)	17–69	0 (0.00)
Midpoint (Week 11)	2.83 (2.71)	0–11	1 (3.33)	29.31 (13.88)	17–73	1 (3.33)
Post-Intervention (Week 17)	2.93 (4.32)	0–20	3 (10.00)	28.33 (13.47)	17–68	3 (10.00)
**Occasion**	**CD-RISC10**			**WEMWBS**		
	***M*** **(*SD*)**	**Range**	**Missing (%)**	***M*** **(*SD*)**	**Range**	**Missing (%)**
Baseline (Week 0)	31.31 (5.97)	16–40	1 (3.33)	50.25 (7.93)	31–61	2 (6.66)
Pre-Intervention (Week 4)	30.27 (5.60)	16–39	0 (0.00)	50.37 (9.20)	30–68	0 (0.00)
Midpoint (Week 11)	31.00 (5.25)	23–40	1 (3.33)	50.48 (7.61)	33–70	1 (3.33)
Post-Intervention (Week 17)	32.04 (5.73)	19–40	3 (10.00)	52.81 (9.74)	33–70	4 (13.33)

CD-RISC10, 10-item Connor–Davidson Resilience Scale; PCL-C, PTSD Checklist, Civilian version; PHQ-9, 9-item Patient Health Questionnaire; WEMWBS, Warwick–Edinburgh Mental Wellbeing Scale.

**Table 4:** Repeated-measures *t*-test results for mental health outcome variables between baseline and pre-intervention (study weeks 0 to 4).

						Hedges’ *g*	
Outcome	*M* Diff. (*SD*)	95% CI	*t*	*p* ^a^	Missing (%)	Point Estimate	95% CI
PHQ-9	0.33 (2.48)	[−0.59, 1.26]	0.74	0.47	0 (0.00)	0.13	[−0.22, 0.49]
PCL-C	0.89 (5.51)	[−1.24, 3.03]	0.86	0.40	2 (6.66)	0.16	[−0.21, 0.53]
CD-RISC10	−1.00 (3.32)	[−2.26, 0.26]	−1.62	0.12	1 (3.33)	−0.30	[−0.66, 0.07]
WEMWBS	−0.46 (4.54)	[−2.23, 1.30]	−0.54	0.59	2 (6.66)	−0.10	[−0.47, 0.27]

CD-RISC10, 10-item Connor–Davidson Resilience Scale; M diff., mean difference; PCL-C, PTSD Checklist, Civilian version; *p*, *p*-value; PHQ-9, 9-item Patient Health Questionnaire; *t*, *t*-value; WEMWBS, Warwick–Edinburgh Mental Wellbeing Scale; 95% CI, 95% confidence interval. All *p*-values presented above are two-tailed. ^a^ Raw *p*-values are presented in this column for each repeated-measures *t*-test. However, a Bonferroni correction for the four comparisons above yields a more conservative *p*-value of 0.013. Comparisons between means should be weighed against this value.

**Table 5:** Repeated-measures *t*-test results for all anthropometric and job-task performance variables between pre- and post-intervention (study weeks 4 to 17).

						Hedges’ *g*	
Variable	*M* Diff. (*SD*)	95% CI	*t*	*p* ^a^	Missing (%)	Point Estimate	95% CI
BW (kg)	−0.72 (2.14)	[−1.72, 0.29]	−1.50	0.15	10 (33.33)	−0.32	[−0.75, 0.12]
LBM (kg)	−0.48 (2.05)	[−1.44, 0.48]	−1.06	0.30	10 (33.33)	−0.23	[−0.65, 0.20]
BMI (kg·m^−2^)	−0.21 (0.63)	[−0.51, 0.08]	−1.51	0.15	10 (33.33)	−0.32	[−0.75, 0.11]
%BF	−0.02 (1.50)	[−0.72, 0.69]	−0.05	0.97	10 (33.33)	−0.01	[−0.43, 0.41]
T-CAC (stations)	2.18 (1.51)	[1.47, 2.88]	6.46	<0.001	10 (33.33)	1.42	[0.79, 2.03]

BMI, body mass index; BW, bodyweight; LBM, lean body mass; lbs., pounds; kg·m^−2^, kilograms per meter squared; *M* diff., mean difference; *p*, *p*-value; *t*, *t*-value; T-CAC, Time-Critical Athlete Challenge; 95% CI, 95% confidence interval; %BF, body fat percentage. T-CAC (stations) reflects the total number of stations participants completed during the 20 min, 8-station circuit workout. All *p*-values presented above are two-tailed. ^a^ Raw *p*-values are presented in this column for each repeated-measures *t*-test. However, a Bonferroni correction for the five comparisons above yields a more conservative *p*-value of 0.01. Comparisons between means should be weighed against this value.

**Table 6:** Repeated-measures *t*-test results for all mental health outcome variables between pre- and post-intervention (study weeks 4 to 17).

						Hedges’ *g*	
Variable	*M* Diff. (*SD*)	95% CI	*t*	*p* ^a^	Missing (%)	Point Estimate	95% CI
PHQ-9	−0.93 (2.90)	[−2.07, 0.22]	−1.66	0.11	3 (10.00)	−0.32	[−0.69, 0.07]
PCL-C	−5.00 (7.40)	[−7.93, −2.07]	−3.51	0.002	3 (10.00)	−0.67	[−1.07, −0.25]
CD-RISC10	2.19 (4.52)	[0.40, 3.97]	2.51	0.02	3 (10.00)	0.48	[0.08, 0.87]
WEMWBS	2.39 (7.10)	[−0.48, 5.25]	1.71	0.10	4 (13.33)	0.33	[−0.06, 0.72]

CD-RISC10, 10-item Connor–Davidson Resilience Scale; *M* diff., mean difference; *p*, *p*-value; PCL-C, PTSD Checklist, Civilian version; PHQ-9, 9-item Patient Health Questionnaire; *t*, *t*-value; WEMWBS, Warwick–Edinburgh Mental Wellbeing Scale; 95% CI, 95% confidence interval. All *p*-values presented above are two-tailed. ^a^ Raw *p*-values are presented in this column for each repeated-measures *t*-test. However, a Bonferroni correction for the four comparisons above yields a more conservative *p*-value of 0.013. Comparisons between means should be weighed against this value.

## Data Availability

The datasets created and/or analyzed for the current study are available from the corresponding author upon reasonable request.

## Supplementary Materials

The following supporting information can be downloaded at https://www.mdpi.com/article/10.3390/ijerph22081227/s1: Table S1: Independent-samples *t*-test results for multiple outcomes between those present (*n* = 20) and absent (*n* = 10) for post-testing; Table S2: Additional exercise types reported by participants during the intervention period; Table S3: Main effects of demographic variables on depressive symptoms centered at pre-intervention (week 4); Table S4: Main effects of demographic variables on post-traumatic stress symptoms centered at pre-intervention (week 4); Table S5: Main effects of demographic variables on psychological resilience centered at pre-intervention (week 4); Table S6: Main effects of demographic variables on mental wellbeing centered at pre-intervention (week 4); Table S7: Main effects of intervention adherence and additional fitness tracking on bodyweight (kg) centered at pre-intervention (week 4); Table S8: Main effects of intervention adherence and additional fitness tracking on lean body mass (kg) centered at pre-intervention (week 4); Table S9: Main effects of intervention adherence and additional fitness tracking on body mass index (kg·m-2 ) centered at pre-intervention (week 4); Table S10: Main effects of intervention adherence and additional fitness tracking on percent body fat centered at pre-intervention (week 4); Table S11: Main effects of intervention adherence and additional fitness tracking on T-CAC performance (stations completed) centered at preintervention (week 4); Table S12: Main effects of intervention adherence and additional fitness tracking on depressive symptoms centered at pre-intervention (week 4); Table S13: Main effects of intervention adherence and additional fitness tracking on post-traumatic stress symptoms centered at pre-intervention (week 4); Table S14: Main effects of intervention adherence and additional fitness tracking on psychological resilience centered at pre-intervention (week 4); Table S15: Main effects of intervention adherence and additional fitness tracking on mental wellbeing centered at pre-intervention (week 4).

## References

[1] Substance Abuse and Mental Health Services Administration Disaster Technical Assistance Center Supplemental Research Bulletin First Responders: Behavioral Health Concerns, Emergency Response, and Trauma; Rockville, MD, 2018. https://www.samhsa.gov/sites/default/files/dtac/supplementalresearchbulletin-firstresponders-may2018.pdf.

[2] Walker A., McKune A., Ferguson S., Pyne D.B., Rattray B. (2016). Chronic Occupational Exposures Can Influence the Rate of PTSD and Depressive Disorders in First Responders and Military Personnel. Extrem. Physiol. Med..

[3] Johnson C.C., Vega L., Kohalmi A.L., Roth J.C., Howell B.R., Van Hasselt V.B. (2020). Enhancing Mental Health Treatment for the Firefighter Population: Understanding Fire Culture, Treatment Barriers, Practice Implications, and Research Directions. Prof. Psychol. Res. Pract..

[4] De Barros V.V., Fernandes Martins L., Saitz R., Rocha Bastos R., Mota Ronzani T. (2013). Mental Health Conditions, Individual and Job Characteristics and Sleep Disturbances among Firefighters. J. Health Psychol..

[5] Stanley I.H., Boffa J.W., Hom M.A., Kimbrel N.A., Joiner T.E. (2017). Differences in Psychiatric Symptoms and Barriers to Mental Health Care between Volunteer and Career Firefighters. Psychiatry Res..

[6] Gulliver S.B., Pennington M.L., Torres V.A., Steffen L.E., Mardikar A., Leto F., Ostiguy W., Zimering R.T., Kimbrel N.A. (2019). Behavioral Health Programs in Fire Service: Surveying Access and Preferences. Psychol. Serv..

[7] Stanley I.H., Hom M.A., Hagan C.R., Joiner T.E. (2015). Career Prevalence and Correlates of Suicidal Thoughts and Behaviors among Firefighters. J. Affect. Disord..

[8] Henderson S.N., van Hasselt V.B., LeDuc T.J., Couwels J. (2016). Firefighter Suicide: Understanding Cultural Challenges for Mental Health Professionals. Prof. Psychol. Res. Pract..

[9] Lee J.H., Lee D., Kim J., Jeon K., Sim M. (2017). Duty-Related Trauma Exposure and Posttraumatic Stress Symptoms in Professional Firefighters. J. Trauma. Stress.

[10] Gulliver S.B., Zimering R.T., Knight J., Morissette S.B., Kamholz B.W., Pennington M.L., Dobani F., Carpenter T.P., Kimbrel N.A., Keane T.M. (2021). A Prospective Study of Firefighters’ PTSD and Depression Symptoms: The First 3 Years of Service. Psychol. Trauma Theory, Res. Pract. Policy.

[11] Wolffe T.A.M., Robinson A., Clinton A., Turrell L., Stec A.A. (2023). Mental Health of UK Firefighters. Sci. Rep..

[12] Brudey C., Park J., Wiaderkiewicz J., Kobayashi I., Mellman T., Marvar P.J. (2015). Autonomic and Inflammatory Consequences of Posttraumatic Stress Disorder and the Link to Cardiovascular Disease. Am. J. Physiol. Regul. Integr. Comp. Physiol..

[13] Michopoulos V., Norrholm S.D., Jovanovic T. (2015). Diagnostic Biomarkers for Posttraumatic Stress Disorder (PTSD): Promising Horizons from Translational Neuroscience Research. Biol. Psychol..

[14] American Psychiatric Association (2013). Diagnostic and Statistical Manual of Mental Disorders.

[15] Blumenthal J.A., Babyak M.A., Moore K.A., Craighead W.E., Herman S., Khatri P., Waugh R., Napolitano M.A., Forman L.M., Appelbaum M. (1999). Effects of Exercise Training on Older Patients with Major Depression. Arch. Intern. Med..

[16] Brosse A.L., Sheets E.S., Lett H.S., Blumenthal J.A. (2002). Exercise and the Treatment of Clinical Depression in Adults: Recent Findings and Future Directions. Sport. Med..

[17] Stathopoulou G., Powers M.B., Berry A.C., Smits J.A.J., Otto M.W. (2006). Exercise Interventions for Mental Health: A Quantitative and Qualitative Review. Clin. Psychol. Sci. Pract..

[18] Rethorst C.D., Wipfli B.M., Landers D.M. (2009). The Antidepressive Effects of Exercise: A Meta-Analysis of Randomized Trials. Sport. Med..

[19] Stanton R., Reaburn P. (2014). Exercise and the Treatment of Depression: A Review of the Exercise Program Variables. J. Sci. Med. Sport.

[20] Schuch F.B., Vancampfort D., Richards J., Rosenbaum S., Ward P.B., Stubbs B. (2016). Exercise as a Treatment for Depression: A Meta-Analysis Adjusting for Publication Bias. J. Psychiatr. Res..

[21] Schuch F.B., Deslandes A.C., Stubbs B., Gosmann N.P., da Silva C.T.B., de Almeida Fleck M.P. (2016). Neurobiological Effects of Exercise on Major Depressive Disorder: A Systematic Review. Neurosci. Biobehav. Rev..

[22] Blumenthal J.A., Smith P.J., Hoffman B.M. (2012). Is Exercise a Viable Treatment for Depression?. ACSM’s Health Fit. J..

[23] Garber C.E., Blissmer B., Deschenes M.R., Franklin B.A., Lamonte M.J., Lee I.M., Nieman D.C., Swain D.P. (2011). Quantity and Quality of Exercise for Developing and Maintaining Cardiorespiratory, Musculoskeletal, and Neuromotor Fitness in Apparently Healthy Adults: Guidance for Prescribing Exercise. Med. Sci. Sports Exerc..

[24] Netz Y. (2017). Is the Comparison between Exercise and Pharmacologic Treatment of Depression in the Clinical Practice Guideline of the American College of Physicians Evidence-Based?. Front. Pharmacol..

[25] Magyari P., Lite R., Killpatrick M.W., Schoffstall J.E. (2018). ACSM’s Resources for the Exercise Physiologist.

[26] Box A.G., Feito Y., Brown C., Heinrich K.M., Petruzzello S.J. (2019). High Intensity Functional Training (HIFT) and Competitions: How Motives Differ by Length of Participation. PLoS ONE.

[27] Wankel L.M. (1993). The Importance of Enjoyment to Adherence and Psychological Benefits from Physical Activity. Int. J. Sport Psychol..

[28] Singh B., Olds T., Curtis R., Dumuid D., Virgara R., Watson A., Szeto K., O’Connor E., Ferguson T., Eglitis E. (2023). Effectiveness of Physical Activity Interventions for Improving Depression, Anxiety and Distress: An Overview of Systematic Reviews. Br. J. Sports Med..

[29] Schuch F.B., Vancampfort D., Firth J., Rosenbaum S., Ward P.B., Silva E.S., Hallgren M., De Leon A.P., Dunn A.L., Deslandes A.C. (2018). Physical Activity and Incident Depression: A Meta-Analysis of Prospective Cohort Studies. Am. J. Psychiatry.

[30] Mikkelsen K., Stojanovska L., Polenakovic M., Bosevski M., Apostolopoulos V. (2017). Exercise and Mental Health. Maturitas.

[31] Sornborger J., Fann A., Serpa J.G., Ventrelle J., R.D.N. M.S., Ming Foynes M., Carleton M., Sherrill A.M., Kao L.K., Jakubovic R. (2017). Integrative Therapy Approaches for Posttraumatic Stress Disorder: A Special Focus on Treating Veterans. Focus (Madison).

[32] Whitworth J.W., Craft L.L., Dunsiger S.I., Ciccolo J.T. (2017). Direct and Indirect Effects of Exercise on Posttraumatic Stress Disorder Symptoms: A Longitudinal Study. Gen. Hosp. Psychiatry.

[33] Hruby A., Lieberman H.R., Smith T.J. (2021). Symptoms of Depression, Anxiety, and Post-Traumatic Stress Disorder and Their Relationship to Health-Related Behaviors in over 12,000 US Military Personnel: Bi-Directional Associations. J. Affect. Disord..

[34] Strohacker K., Fazzino D., Breslin W.L., Xu X. (2015). The Use of Periodization in Exercise Prescriptions for Inactive Adults: A Systematic Review. Prev. Med. Reports.

[35] McKeon G., Steel Z., Wells R., Newby J., Hadzi-Pavlovic D., Vancampfort D., Rosenbaum S. (2021). A Mental Health–Informed Physical Activity Intervention for First Responders and Their Partners Delivered Using Facebook: Mixed Methods Pilot Study. JMIR Form. Res..

[36] Rosenbaum S., Sherrington C., Tiedemann A. (2015). Exercise Augmentation Compared with Usual Care for Post-Traumatic Stress Disorder: A Randomized Controlled Trial. Acta Psychiatr. Scand..

[37] Ashdown-Franks G., Firth J., Carney R., Carvalho A.F., Hallgren M., Koyanagi A., Rosenbaum S., Schuch F.B., Smith L., Solmi M. (2020). Exercise as Medicine for Mental and Substance Use Disorders: A Meta-Review of the Benefits for Neuropsychiatric and Cognitive Outcomes. Sport. Med..

[38] Rosenbaum S., Vancampfort D., Steel Z., Newby J., Ward P.B., Stubbs B. (2015). Physical Activity in the Treatment of Post-Traumatic Stress Disorder: A Systematic Review and Meta-Analysis. Psychiatry Res..

[39] Feito Y., Heinrich K.M., Butcher S.J., Poston W.S.C. (2018). High-Intensity Functional Training (HIFT): Definition and Research Implications for Improved Fitness. Sports.

[40] Bycura D.K., Repka C.P., Santos A.C., Lopez N.V. (2019). Training Implications for Firefighters through Objective Measurement of the Physiological Demands of Firefighter Job Tasks. Am. J. Biomed. Sci. Res..

[41] Haddock C.K., Poston W.S.C., Heinrich K.M., Jahnke S.A., Jitnarin N. (2016). The Benefits of High Intensity Functional Training (HIFT) Fitness Programs for Military Personnel. Mil. Med..

[42] Smith D.L. (2011). Firefighter Fitness: Improving Performance and Preventing Injuries and Fatalities. Curr. Sports Med. Rep..

[43] Bycura D.K., Dmitrieva N.O., Santos A.C., Waugh K.L., Ritchey K.M. (2019). Efficacy of a Goal Setting and Implementation Planning Intervention on Firefighters’ Cardiorespiratory Fitness. J. Strength Cond. Res..

[44] Smith B.W., Ortiz J.A., Steffen L.E., Tooley E.M., Wiggins K.T., Yeater E.A., Montoya J.D., Bernard M.L. (2011). Mindfulness Is Associated with Fewer PTSD Symptoms, Depressive Symptoms, Physical Symptoms, and Alcohol Problems in Urban Firefighters. J. Consult. Clin. Psychol..

[45] Skeffington P.M., Rees C.S., Mazzucchelli T.G., Kane R.T. (2016). The Primary Prevention of PTSD in Firefighters: Preliminary Results of an RCT with 12-Month Follow-Up. PLoS ONE.

[46] Boffa J.W., Stanley I.H., Hom M.A., Norr A.M., Joiner T.E., Schmidt N.B. (2017). PTSD Symptoms and Suicidal Thoughts and Behaviors among Firefighters. J. Psychiatr. Res..

[47] Haglund M.E.M., Nestadt P.S., Cooper N.S., Southwick S.M., Charney D.S. (2007). Psychobiological Mechanisms of Resilience: Relevance to Prevention and Treatment of Stress-Related Psychopathology. Dev. Psychopathol..

[48] Schäfer S.K., Sopp M.R., Staginnus M., Lass-Hennemann J., Michael T. (2020). Correlates of Mental Health in Occupations at Risk for Traumatization: A Cross-Sectional Study. BMC Psychiatry.

[49] Krakauer R.L., Stelnicki A.M., Carleton R.N. (2020). Examining Mental Health Knowledge, Stigma, and Service Use Intentions Among Public Safety Personnel. Front. Psychol..

[50] Joyce S., Shand F., Bryant R.A., Lal T.J., Harvey S.B. (2018). Mindfulness-Based Resilience Training in the Workplace: Pilot Study of the Internet-Based Resilience@Work (RAW) Mindfulness Program. J. Med. Internet Res..

[51] MacMillan F., Kolt G.S., Le A., George E.S. (2021). Systematic Review of Randomised Control Trial Health Promotion Intervention Studies in the Fire Services: Study Characteristics, Intervention Design and Impacts on Health. Occup. Environ. Med..

[52] Kyron M.J., Rees C.S., Lawrence D., Carleton R.N., McEvoy P.M. (2021). Prospective Risk and Protective Factors for Psychopathology and Wellbeing in Civilian Emergency Services Personnel: A Systematic Review. J. Affect. Disord..

[53] Kshtriya S., Kobezak H.M., Popok P., Lawrence J., Lowe S.R. (2020). Social Support as a Mediator of Occupational Stressors and Mental Health Outcomes in First Responders. J. Community Psychol..

[54] Bartlett B.A., Smith L.J., Tran J.K., Vujanovic A.A. (2018). Understanding Mental Health among Military Veterans in the Fire Service. Psychiatry Res..

[55] Carleton R.N., Afifi T.O., Turner S., Taillieu T., Vaughan A.D., Anderson G.S., Ricciardelli R., MacPhee R.S., Cramm H.A., Czarnuch S. (2020). Mental Health Training, Attitudes toward Support, and Screening Positive for Mental Disorders. Cogn. Behav. Ther..

[56] Deady M., Peters D., Lang H., Calvo R., Glozier N., Christensen H., Harvey S.B. (2017). Designing Smartphone Mental Health Applications for Emergency Service Workers. Occup. Med..

[57] Denkova E., Zanesco A.P., Rogers S.L., Jha A.P. (2020). Is Resilience Trainable? An Initial Study Comparing Mindfulness and Relaxation Training in Firefighters. Psychiatry Res..

[58] Onyedire N.G., Ekoh A.T., Chukwuorji J.B.C., Ifeagwazi C.M. (2017). Posttraumatic Stress Disorder (PTSD) Symptoms among Firefighters: Roles of Resilience and Locus of Control. J. Workplace Behav. Health.

[59] Mao X., Fung O.W.M., Hu X., Loke A.Y. (2018). Psychological Impacts of Disaster on Rescue Workers: A Review of the Literature. Int. J. Disaster Risk Reduct..

[60] Stanley I.H., Hom M.A., Chu C., Dougherty S.P., Gallyer A.J., Spencer-Thomas S., Shelef L., Fruchter E., Comtois K.A., Gutierrez P.M. (2019). Perceptions of Belongingness and Social Support Attenuate PTSD Symptom Severity Among Firefighters: A Multistudy Investigation. Psychol. Serv..

[61] Meyer E.C., Zimering R.T., Knight J., Morissette S.B., Kamholz B.W., Coe E., Carpenter T.P., Keane T.M., Kimbrel N.A., Gulliver S.B. (2021). Negative Emotionality Interacts with Trauma Exposure to Prospectively Predict Posttraumatic Stress Disorder Symptoms During Firefighters’ First 3 Years of Service. J. Trauma. Stress.

[62] Miller A., Unruh L., Wharton T., Liu X., Zhang N. (2018). Individual and Organizational Factors Associated with Professional Quality of Life in Florida Fire Personnel. J. Emerg. Manag..

[63] Witt M., Stelcer B., Czarnecka-Iwańczuk M. (2018). Stress Coping Styles in Firemen Exposed to Severe Stress. Psychiatr. Pol..

[64] Counson I., Hosemans D., Lal T.J., Mott B., Harvey S.B., Joyce S. (2019). Mental Health and Mindfulness amongst Australian Fire Fighters. BMC Psychol..

[65] Hegberg N.J., Hayes J.P., Hayes S.M. (2019). Exercise Intervention in PTSD: A Narrative Review and Rationale for Implementation. Front. Psychiatry.

[66] Whitworth J.W., Ciccolo J.T. (2016). Exercise and Post-Traumatic Stress Disorder in Military Veterans: A Systematic Review. Mil. Med..

[67] Robertson I.T., Cooper C.L., Sarkar M., Curran T. (2015). Resilience Training in the Workplace from 2003 to 2014: A Systematic Review. J. Occup. Organ. Psychol..

[68] Leppin A.L., Bora P.R., Tilburt J.C., Gionfriddo M.R., Zeballos-Palacios C., Dulohery M.M., Sood A., Erwin P.J., Brito J.P., Boehmer K.R. (2014). The Efficacy of Resiliency Training Programs: A Systematic Review and Meta-Analysis of Randomized Trials. PLoS ONE.

[69] Hollerbach B.S., Jahnke S.A., Poston W.S.C., Harms C.A., Heinrich K.M. (2019). Examining a Novel Firefighter Exercise Training Program on Simulated Fire Ground Test Performance, Cardiorespiratory Endurance, and Strength: A Pilot Investigation. J. Occup. Med. Toxicol..

[70] Day R.S., Jahnke S.A., Haddock C.K., Kaipust C.M., Jitnarin N., Poston W.S.C. (2019). Occupationally Tailored, Web-Based, Nutrition and Physical Activity Program for Firefighters: Cluster Randomized Trial and Weight Outcome. J. Occup. Environ. Med..

[71] The First Twenty The First 20 Academy. https://www.thefirsttwenty.org/programming/.

[72] Harris P.A., Taylor R., Thielke R., Payne J., Gonzalez N., Conde J.G. (2009). Research Electronic Data Capture (REDCap)-A Metadata-Driven Methodology and Workflow Process for Providing Translational Research Informatics Support. J. Biomed. Inform..

[73] Harris P.A., Taylor R., Minor B.L., Elliott V., Fernandez M., O’Neal L., McLeod L., Delacqua G., Delacqua F., Kirby J. (2019). The REDCap Consortium: Building an International Community of Software Platform Partners. J. Biomed. Inform..

[74] International Association of Fire Fighters (2007). The Fire Service Joint Labor Management Wellness-Fitness Initiative Candidate Physical Ability Test.

[75] Spitzer R.L., Kroenke K., Williams J.B.W. (1999). Validation and Utility of a Self-Report Version of PRIME-MD: The PHQ Primary Care Study. Primary Care Evaluation of Mental Disorders. Patient Health Questionnaire. JAMA.

[76] Blanchard E.B., Jones-Alexander J., Buckley T.C., Forneris C.A. (1996). Psychometric Properties of the PTSD Checklist (PCL). Behav. Res. Ther..

[77] Campbell-Sills L., Stein M.B. (2007). Psychometric Analysis and Refinement of the Connor–Davidson Resilience Scale (CD-RISC): Validation of a 10-Item Measure of Resilience. J. Trauma. Stress.

[78] Tennant R., Hiller L., Fishwick R., Platt S., Joseph S., Weich S., Parkinson J., Secker J., Stewart-Brown S. (2007). The Warwick-Edinburgh Mental Well-Being Scale (WEMWBS): Development and UK Validation. Health Qual. Life Outcomes.

[79] Kroenke K., Spitzer R.L., Williams J.W. (2001). The Patient Health Questionnaire PHQ-9: Validity of a Brief Depression Severity Measure. J. Gen. Intern. Med..

[80] Lang A.J., Laffaye C., Satz L.E., Dresselhaus T.R., Stein M.B. (2003). Sensitivity and Specificity of the PTSD Checklist in Detecting PTSD in Female Veterans in Primary Care. J. Trauma. Stress.

[81] Moshier S.J., Lee D.J., Bovin M.J., Gauthier G., Zax A., Rosen R.C., Keane T.M., Marx B.P. (2019). An Empirical Crosswalk for the PTSD Checklist: Translating DSM-IV to DSM-5 Using a Veteran Sample. J. Trauma. Stress.

[82] Salisu I., Hashim N. (2017). A Critical Review of Scales Used in Resilience Research. IOSR J. Bus. Manag..

[83] Davidson J.R.T. (2020). Connor-Davidson Resilience Scale (CD-RISC) © Manual.

[84] Connor K.M., Davidson J.R.T. (2020). Scoring and Interpretation of the Connor-Davidson Resilience Scale (CD-RISC©).

[85] Connor K.M., Davidson J.R.T. The Connor-Davidson Resilience Scale. http://www.connordavidson-resiliencescale.com/about.php.

[86] Stewart-Brown S., Tennant A., Tennant R., Platt S., Parkinson J., Weich S. (2009). Internal Construct Validity of the Warwick-Edinburgh Mental Well-Being Scale (WEMWBS): A Rasch Analysis Using Data from the Scottish Health Education Population Survey. Health Qual. Life Outcomes.

[87] Maheswaran H., Weich S., Powell J., Stewart-Brown S. (2012). Evaluating the Responsiveness of the Warwick Edinburgh Mental Well-Being Scale (WEMWBS): Group and Individual Level Analysis. Health Qual. Life Outcomes.

[88] (2015). Warwick-Edinburgh Mental Well-Being Scale (WEMWBS): User Guide—Version 2.

[89] Haff G.G., Triplett N.T. (2016). Essentials of Strength Training and Conditioning.

[90] Microsoft Corporation (2018). Microsoft Excel.

[91] IBM Corp (2021). IBM SPSS Statistics for Windows.

[92] Glen S. Hedges’ g: Definition, Formula. Statistics How To.com: Elementary Statistics for the Rest of Us!.

[93] Brydges C.R. (2019). Effect Size Guidelines, Sample Size Calculations, and Statistical Power in Gerontology. Innov. Aging.

[94] Quintana D.S. (2017). Statistical Considerations for Reporting and Planning Heart Rate Variability Case-Control Studies. Psychophysiology.

[95] Lovakov A., Agadullina E.R. (2021). Empirically Derived Guidelines for Effect Size Interpretation in Social Psychology. Eur. J. Soc. Psychol..

[96] National Institute of Standards and Technology Hedge’s g Statistic. https://www.itl.nist.gov/div898/software/dataplot/refman2/auxillar/hedgeg.htm.

[97] Fritz C.O., Morris P.E., Richler J.J. (2012). Effect Size Estimates: Current Use, Calculations, and Interpretation. J. Exp. Psychol. Gen..

[98] Faul F., Erdfelder E., Lang A.-G., Buchner A. (2007). G*Power 3: A Flexible Statistical Power Analysis Program for the Social, Behavioral, and Biomedical Sciences. Behav. Res. Methods.

[99] Faul F., Erdfelder E., Buchner A., Lang A.-G. (2009). Statistical Power Analyses Using G*Power 3.1: Tests for Correlation and Regression Analyses. Behav. Res. Methods.

[100] (2015). Etymologia: Bonferroni Correction. Emerg. Infect. Dis..

[101] SAS Institute Inc (2016). SAS for Windows.

[102] Singer J.D., Willett J.B. (2003). Applied Longitudinal Data Analysis: Modeling Change and Event Occurrence.

[103] Hoffman L. (2015). Longitudinal Analysis: Modeling Within-Person Fluctuation and Change.

[104] Borg G. (1998). Borg’s Perceived Exertion and Pain Scales.

[105] Lakens D. (2013). Calculating and Reporting Effect Sizes to Facilitate Cumulative Science: A Practical Primer for t-Tests and ANOVAs. Front. Psychol..

[106] Taylor J.M., Alanazi S. (2023). Cohen’s and Hedges’ G. J. Nurs. Educ..

[107] National Center for PTSD Using the PTSD Checklist for DSM-IV (PCL).

[108] Prime-md (2005). Patient Health Questionnaire (PHQ-9).

[109] Corthésy-Blondin L., Genest C., Dargis L., Bardon C., Mishara B.L. (2022). Reducing the Impacts of Exposure to Potentially Traumatic Events on the Mental Health of Public Safety Personnel: A Rapid Systematic Scoping Review. Psychol. Serv..

[110] Jahnke S.A., Gist R., Poston W.S.C., Haddock C.K. (2014). Behavioral Health Interventions in the Fire Service: Stories from the Firehouse. J. Workplace Behav. Health.

[111] Lentz L., Smith-Macdonald L., Malloy D.C., Anderson G.S., Beshai S., Ricciardelli R., Brémault-Phillips S., Carleton R.N. (2022). A Qualitative Analysis of the Mental Health Training and Educational Needs of Firefighters, Paramedics, and Public Safety Communicators in Canada. Int. J. Environ. Res. Public Health.

[112] Tabachnick B.G., Fidell L.S. (2013). Using Multivariate Statistics.

[113] Sempf F., Thienes G. (2022). High-Intensity Functional Training for Firefighters. Strength Cond. J..

[114] Stratton E., Lampit A., Choi I., Calvo R.A., Harvey S.B., Glozier N. (2017). Effectiveness of EHealth Interventions for Reducing Mental Health Conditions in Employees: A Systematic Review and Meta-Analysis. PLoS ONE.

[115] Igboanugo S., Bigelow P.L., Mielke J.G. (2021). Health Outcomes of Psychosocial Stress within Firefighters: A Systematic Review of the Research Landscape. J. Occup. Health.

[116] Hobfoll S.E., Halbesleben J., Neveu J.-P., Westman M. (2018). Conservation of Resources in the Organizational Context: The Reality of Resources and Their Consequences. Annu. Rev. Organ. Psychol. Organ. Behav..

[117] Sattler D.N., Preston A.J., Kaiser C.F., Olivera V.E., Valdez J., Schlueter S. (2002). Hurricane Georges: A Cross-National Study Examining Preparedness, Resource Loss, and Psychological Distress in the U.S. Virgin Islands, Puerto Rico, Dominican Republic, and the United States. J. Trauma. Stress.

[118] Sattler D.N., Boyd B., Kirsch J. (2014). Trauma-Exposed Firefighters: Relationships among Posttraumatic Growth, Posttraumatic Stress, Resource Availability, Coping and Critical Incident Stress Debriefing Experience. Stress Health.

[119] Joyce S., Shand F., Lal T.J., Mott B., Bryant R.A., Harvey S.B. (2019). Resilience@Work Mindfulness Program: Results from a Cluster Randomized Controlled Trial with First Responders. J. Med. Internet Res..

[120] Christopher M.S., Hunsinger M., Goerling L.R.J., Bowen S., Rogers B.S., Gross C.R., Dapolonia E., Pruessner J.C. (2018). Mindfulness-Based Resilience Training to Reduce Health Risk, Stress Reactivity, and Aggression among Law Enforcement Officers: A Feasibility and Preliminary Efficacy Trial. Psychiatry Res..

[121] Arnetz B.B., Arble E., Backman L., Lynch A., Lublin A. (2013). Assessment of a Prevention Program for Work-Related Stress among Urban Police Officers. Int. Arch. Occup. Environ. Health.

[122] Pace T.W.W., Zeiders K.H., Cook S.H., Sarsar E.D., Hoyt L.T., Mirin N.L., Wood E.P., Tatar R., Davidson R.J. (2022). Feasibility, Acceptability, and Preliminary Efficacy of an App-Based Meditation Intervention to Decrease Firefighter Psychological Distress and Burnout: A One-Group Pilot Study. JMIR Form. Res..

[123] Sharp P., Caperchione C.M., Brown G.A., Stadnyk A., Marin E., Hulin B., Wade J., Mott B., Gabriel M., Impellizzeri F. (2023). A Pragmatic Strength and Conditioning Intervention for Firefighters: Feasibility of the Tactical Athlete Resilience Program (TARP). Health Promot. J. Aust..

[124] Heydari A., Ostadtaghizadeh A., Khorasani-Zavareh D., Ardalan A., Ebadi A., Mohammadfam I., Shafaei H. (2022). Building Resilience in Firefighters: A Systematic Review. Iran. J. Public Health.

[125] Alexander L., Cooper K. (2019). Vocational Rehabilitation for Emergency Services Personnel: A Scoping Review. JBI Evid. Synth..

[126] Elliot D.L., Goldberg L., Duncan T.E., Kuehl K.S., Moe E.L., Rosemary Breger M.K., DeFrancesco C.L., Denise Ernst R.B., Stevens V.J. (2004). The PHLAME Firefighters’ Study: Feasibility and Findings. Am. J. Health Behav..

[127] Norris R., Carroll D., Cochrane R. (1990). The Effects of Aerobic and Anaerobic Training on Fitness, Blood Pressure, and Psychological Stress and Well-Being. J. Psychosom. Res..

[128] Norvell N., Belles D. (1993). Psychological and Physical Benefits of Circuit Weight Training in Law Enforcement Personnel. J. Consult. Clin. Psychol..

[129] Lan F.Y., Scheibler C., Hershey M.S., Romero-Cabrera J.L., Gaviola G.C., Yiannakou I., Fernandez-Montero A., Christophi C.A., Christiani D.C., Sotos-Prieto M. (2022). Effects of a Healthy Lifestyle Intervention and COVID-19-Adjusted Training Curriculum on Firefighter Recruits. Sci. Rep..

[130] Fraess-Phillips A., Wagner S., Harris R.L. (2017). Firefighters and Traumatic Stress: A Review. Int. J. Emerg. Serv..

[131] Sommerfeld A., Wagner S.L., Harder H.G., Schmidt G. (2017). Behavioral Health and Firefighters: An Intervention and Interviews with Canadian Firefighters. J. Loss Trauma.

[132] Laureys V., Easton M. (2020). Resilience of Firefighters Exposed to Potentially Traumatic Events: A Literature Review. Int. J. Emerg. Serv..

