# Supplementary material for: Effectiveness of a Mind–Body Intervention at Improving Mental Health and Performance Among Career Firefighters
Source: Int J Environ Res Public Health. 2025 Aug 6;22(8):1227. doi: 10.3390/ijerph22081227 (PMC12386839; doi:10.3390/ijerph22081227)
Supplement: Supplementary file 1 [file ijerph-22-01227-s001.zip › Table S5 Main effects of demographic variables on psychological resilience centered at pre-intervention (week 4).pdf]

Table S5. Main effects of demographic variables on psychological resilience centered at pre-intervention (week 4).

Parameter	Model 1 *B* (*SE*)	Model 2 *B* (*SE*)	Model 3 *B* (*SE*)	Model 4 *B* (*SE*)	Model 5 *B* (*SE*)	Model 6 *B* (*SE*)	Model 7 *B* (*SE*)	Model 8 *B* (*SE*)	Model 9 *B* (*SE*)	Model 10 *B* (*SE*)
**Fixed Effects**										
Intercept	31.18‡ (0.90)	30.20‡ (0.97)	30.26‡ (0.65)	30.25‡ (0.66)	30.63‡ (0.86)	30.63‡ (0.89)	30.29‡ (0.65)	29.99‡ (1.53)	28.61‡ (1.22)	30.33‡ (0.68)
Baseline score$_{MC}$[a]			0.70‡ (0.09)	0.71‡ (0.10)	0.69‡ (0.09)	0.69‡ (0.09)	0.68‡ (0.10)	0.70‡ (0.09)	0.73‡ (0.09)	0.70‡ (0.09)
Age$_{MC}$ (years)[b]				0.01 (0.08)						
Education[c]					-0.73 (1.09)					
Rank[d]						-0.66 (1.11)				
Years of service$_{MC}$[e]							-0.04 (0.07)			
Race and ethnicity[f]								0.31 (1.60)		
Relationship status[g]									2.08 (1.31)	
Sex[h]										-0.72 (1.77)
Growth		0.14† (0.05)	0.14* (0.05)	0.14* (0.05)	0.14* (0.05)	0.14* (0.05)	0.14* (0.05)	0.14* (0.05)	0.14* (0.05)	0.14* (0.05)
**Random Effects**										
Intercept	21.55‡ (6.32)	21.97‡ (6.34)	5.86† (2.24)	5.85† (2.24)	5.73† (2.21)	5.76† (2.21)	5.75† (2.21)	5.85† (2.24)	5.17* (2.23)	5.81† (2.07)
Residual	8.17‡ (1.54)	7.20‡ (1.36)	7.40‡ (1.41)	7.40‡ (1.41)	7.40‡ (1.41)	7.40‡ (1.41)	7.40‡ (1.41)	7.40‡ (1.41)	7.41‡ (1.41)	7.40‡ (1.42)
**Pseudo R^2**										

		.0198	.5683	.5685	.5725	.5716	.5717	.5685	.5916	.5699
**Model Deviance**										
–2 log-likelihood	488.9	481.9	435.9	435.9	435.5	435.5	435.5	435.9	433.5	435.7
AIC	494.9	489.9	445.9	447.9	447.5	447.5	447.5	447.9	445.5	447.7
BIC	499.1	495.5	452.7	456.1	455.7	455.7	455.7	456.1	453.7	455.9

Note: AIC, Akaike Information Criterion; BIC, Bayesian Information Criterion; *SE*, standard error. Psychological resilience was measured using the 10-item Connor-Davidson Resilience Scale (CD-RISC10; range = 0-40).

* indicates two-tailed $p < .05$, † indicates two-tailed $p < .01$, ‡ indicates two-tailed $p < .001$.

[a] For mean-centered psychological resilience at baseline, the model value of 0 = 31.31 (*SD* = 5.96). Baseline scores were collected four weeks prior to pre-intervention testing.

[b] Participants' age was centered at 39 years (*M* = 39.70, *SD* = 7.71).

[c] For education level, model values included 0 = Some college but no degree (*n* = 2) or Associate degree (*n* = 13); and 1 = Bachelor degree (*n* = 13) or Graduate degree (*n* = 2).

[d] For fire department rank, model values included 0 = Firefighter (*n* = 8) or Engineer (*n* = 6); and 1 = Captain (*n* = 10) or Battalion Chief (*n* = 6).

[e] Participants' years in the fire service was centered at 15 years (*M* = 15.43, *SD* = 8.37).

[f] Participants' responses for race and ethnicity were combined into one common model predictor. Model values included 0 = (Race: White [*n* = 1], Other [*n* = 1], Don't know [*n* = 1], or Prefer not to say [*n* = 1]; Ethnicity: Hispanic [*n* = 3] or Prefer not to say [*n* = 1]); and 1 = (Race: White [*n* = 26]; Ethnicity: Not Hispanic [*n* = 26]).

[g] For participants' relationship status, model values included 0 = Single (*n* = 3), In a relationship (*n* = 2), or Divorced (*n* = 1); and 1 = Married (*n* = 24).

[h] Model values included 0 = Male (*n* = 27), and 1 = Female (*n* = 3).
