# Supplementary material for: Effectiveness of a Mind–Body Intervention at Improving Mental Health and Performance Among Career Firefighters
Source: Int J Environ Res Public Health. 2025 Aug 6;22(8):1227. doi: 10.3390/ijerph22081227 (PMC12386839; doi:10.3390/ijerph22081227)
Supplement: Supplementary file 1 [file ijerph-22-01227-s001.zip › Table S11 Main effects of intervention adherence and additional fitness tracking on T-CAC performance centered at pre-intervention (week 4).pdf]

Table S11. Main effects of intervention adherence and additional fitness tracking on T-CAC performance (stations completed) centered at pre-intervention (week 4).

Parameter	Model 1 *B* (*SE*)	Model 2 *B* (*SE*)	Model 3 *B* (*SE*)	Model 4 *B* (*SE*)	Model 5 *B* (*SE*)	Model 6 *B* (*SE*)	Model 7 *B* (*SE*)	Model 8 *B* (*SE*)	Model 9 *B* (*SE*)	Model 10 *B* (*SE*)	Model 11 *B* (*SE*)
**Fixed Effects**											
Intercept	22.93‡ (0.73)	22.11‡ (0.71)	22.11‡ (0.64)	22.11‡ (0.64)	22.11‡ (0.66)	22.11‡ (0.63)	22.11‡ (0.63)	22.17‡ (0.63)	22.11‡ (0.68)	22.11‡ (0.67)	22.29‡ (0.70)
Combined adherence$_{STD}$[a]			1.75* (0.65)	1.80† (0.65)	1.81 (1.02)						
Combined adherence$_{STD}$ × Growth interaction				-0.04 (0.05)	-0.04 (0.05)						
HIFT adherence$_{STD}$[b]						1.89† (0.64)	1.96† (0.64)	1.81* (0.79)			
HIFT adherence$_{STD}$ × Growth interaction							-0.07 (0.05)	-0.07 (0.05)			
RES adherence$_{STD}$[c]									1.31 (0.68)	1.32 (0.69)	0.50 (1.17)
RES adherence$_{STD}$ × Growth interaction										-0.01 (0.05)	-0.00 (0.05)
Additional workouts$_{MCd}$					-0.08 (0.28)			-0.08 (0.26)			0.04 (0.29)
Additional minutes of exercise$_{MC}$[e]					0.00 (0.00)			0.00 (0.00)			0.00 (0.00)
RPE of additional workouts$_{MC}$[f]					-0.07 (0.31)			-0.09 (0.30)			-0.08 (0.33)
Growth		0.18‡ (0.03)	0.17‡ (0.03)	0.19‡ (0.03)	0.19‡ (0.03)	0.17‡ (0.03)	0.20‡ (0.03)	0.20‡ (0.03)	0.18‡ (0.03)	0.18‡ (0.03)	0.18‡ (0.03)
**Random Effects**											

Intercept	13.86† (4.29)	14.22‡ (3.87)	11.38‡ (3.11)	11.27‡ (3.09)	11.04‡ (3.07)	10.91‡ (2.99)	10.82‡ (2.96)	10.44‡ (2.90)	12.64‡ (3.45)	12.62‡ (3.45)	12.18‡ (3.39)
Residual	3.50‡ (1.12)	1.04‡ (0.33)	1.03‡ (0.32)	1.01‡ (0.32)	1.01‡ (0.32)	1.03‡ (0.32)	0.95‡ (0.30)	0.95‡ (0.30)	1.03‡ (0.33)	1.03‡ (0.33)	1.03‡ (0.33)
**Pseudo R^2**											
		.1371	.2319	.2403	.1929	.2604	.2705	.2323	.1774	.1790	.1532
**Model Deviance**											
–2 log-likelihood	264.3	237.5	231.0	230.4	224.5	229.8	228.0	221.7	234.1	234.0	227.6
AIC	270.3	245.5	241.0	242.4	242.5	239.8	240.0	239.7	244.1	246.0	245.6
BIC	274.5	251.1	248.0	250.8	254.8	246.8	248.4	252.0	251.1	254.4	257.9

Note. AIC, Akaike Information Criterion; BIC, Bayesian Information Criterion; *SE*, standard error.

* indicates two-tailed $p < .05$, † indicates two-tailed $p < .01$, ‡ indicates two-tailed $p < .001$.

[a] Standardized combined adherence was calculated by first adding participants' total HIFT workouts and RES practices completed before subtracting the grand mean ($M = 69.90$, $SD = 16.12$). This value was then divided by the standard deviation of the grand mean. Outliers were not removed to best characterize effects on the full availability of participant data.

[b] Standardized HIFT adherence was calculated by subtracting the grand mean ($M = 28.13$, $SD = 8.93$) from participants' total HIFT workouts completed. This value was then divided by the standard deviation of the grand mean. Outliers were not removed.

[c] Standardized RES adherence was calculated by subtracting the grand mean ($M = 41.77$, $SD = 8.71$) from participants' total RES workouts completed. This value was then divided by the standard deviation of the grand mean. Outliers were not removed.

[d] For mean-centered additional workouts completed each week during the intervention, the model value of 0 = 3.57 ($SD = 2.49$). Outliers were not removed.

[e] For mean-centered additional minutes of exercise completed each week during the intervention, the model value of 0 = 238.04 ($SD = 180.81$). Outliers were not removed.

[f] For mean-centered RPE of additional workouts completed each week during the intervention, the model value of 0 = 13.49 ($SD = 2.05$). Outliers were not removed.
