# Supplementary material for: Effectiveness of a Mind–Body Intervention at Improving Mental Health and Performance Among Career Firefighters
Source: Int J Environ Res Public Health. 2025 Aug 6;22(8):1227. doi: 10.3390/ijerph22081227 (PMC12386839; doi:10.3390/ijerph22081227)
Supplement: Supplementary file 1 [file ijerph-22-01227-s001.zip › Table S15 Main effects of intervention adherence and additional fitness tracking on mental wellbeing centered at pre-intervention (week 4).pdf]

**Table S15.** Main effects of intervention adherence and additional fitness tracking on mental wellbeing centered at pre-intervention (week 4).

| Parameter | Model 1 *B* (*SE*) | Model 2 *B* (*SE*) | Model 3 *B* (*SE*) | Model 4 *B* (*SE*) | Model 5 *B* (*SE*) | Model 6 *B* (*SE*) | Model 7 *B* (*SE*) | Model 8 *B* (*SE*) | Model 9 *B* (*SE*) | Model 10 *B* (*SE*) | Model 11 *B* (*SE*) | Model 12 *B* (*SE*) |
|---|---|---|---|---|---|---|---|---|---|---|---|---|
| **Fixed Effects** | | | | | | | | | | | | |
| Intercept | 51.24‡ (1.48) | 50.19‡ (1.59) | 49.61‡ (1.10) | 49.61‡ (1.10) | 49.61‡ (1.10) | 49.34‡ (1.10) | 49.61‡ (1.09) | 49.61‡ (1.09) | 49.48‡ (1.08) | 49.61‡ (1.10) | 49.61‡ (1.10) | 49.35‡ (1.11) |
| Baseline score$_{MC}$[a] | | | 0.83‡ (0.12) | 0.83‡ (0.12) | 0.83‡ (0.12) | 0.85‡ (0.13) | 0.83‡ (0.12) | 0.83‡ (0.12) | 0.84‡ (0.13) | 0.83‡ (0.12) | 0.83‡ (0.12) | 0.87‡ (0.13) |
| Combined adherence$_{STD}$[b] | | | | 0.37 (0.95) | 0.46 (1.10) | 1.85 (1.61) | | | | | | |
| Combined adherence$_{STD}$ × Growth interaction | | | | | -0.01 (0.09) | -0.11 (0.13) | | | | | | |
| HIFT adherence$_{STD}$[c] | | | | | | | 0.67 (0.93) | 0.66 (1.08) | 1.36 (1.29) | | | |
| HIFT adherence$_{STD}$ × Growth interaction | | | | | | | | 0.00 (0.08) | -0.04 (0.10) | | | |
| RES adherence$_{STD}$[d] | | | | | | | | | | -0.03 (0.98) | 0.15 (1.12) | 1.54 (1.78) |
| RES adherence$_{STD}$ × Growth interaction | | | | | | | | | | | -0.03 (0.09) | -0.18 (0.15) |
| Additional workouts$_{MC}$[e] | | | | | | -0.02 (0.38) | | | -0.01 (0.36) | | | 0.06 (0.38) |
| Additional minutes of exercise$_{MC}$[f] | | | | | | 0.01 (0.01) | | | 0.01 (0.01) | | | 0.01 (0.01) |
| RPE of additional workouts$_{MC}$[g] | | | | | | 0.78 (0.43) | | | 0.77 (0.43) | | | 0.78 (0.43) |
| Growth | | 0.16 (0.09) | 0.21* (0.09) | 0.21* (0.09) | 0.21* (0.09) | 0.24* (0.09) | 0.21* (0.09) | 0.21* (0.09) | 0.22* (0.09) | 0.21* (0.09) | 0.21* (0.09) | 0.26† (0.10) |
| **Random Effects** | | | | | | | | | | | | |

| | | | | | | | | | | | | |
|---|---|---|---|---|---|---|---|---|---|---|---|---|
| Intercept | 57.89‡ (17.11) | 58.06‡ (17.04) | 18.37† (6.93) | 18.25† (6.89) | 18.21† (6.88) | 14.75* (6.02) | 17.93† (6.80) | 17.93† (6.81) | 14.63* (6.01) | 18.37† (6.93) | 18.31† (6.91) | 15.06* (6.11) |
| Residual | 21.32‡ (4.08) | 20.21‡ (3.87) | 17.68‡ (3.54) | 17.67‡ (3.54) | 17.68‡ (3.55) | 17.81‡ (3.60) | 17.67‡ (3.54) | 17.67‡ (3.54) | 17.93‡ (3.63) | 17.68‡ (3.55) | 17.67‡ (3.54) | 17.62‡ (3.57) |
| **Pseudo $R^2$** | | | | | | | | | | | | |
| | | .0130 | .5476 | .5489 | .5492 | .5848 | .5528 | .5528 | .5854 | .5477 | .5485 | .5845 |
| **Model Deviance** | | | | | | | | | | | | |
| –2 log-likelihood | 565.9 | 562.9 | 489.3 | 489.1 | 489.1 | 472.9 | 488.8 | 488.8 | 473.1 | 489.3 | 489.2 | 472.6 |
| AIC | 571.9 | 570.9 | 499.3 | 501.1 | 503.1 | 492.9 | 500.8 | 502.8 | 493.1 | 501.3 | 503.2 | 492.6 |
| BIC | 576.1 | 576.5 | 505.9 | 509.1 | 512.4 | 505.8 | 508.7 | 512.1 | 506.1 | 509.3 | 512.5 | 505.6 |

*Note.* AIC, Akaike Information Criterion; BIC, Bayesian Information Criterion; *SE*, standard error.

* indicates two-tailed $p < .05$, † indicates two-tailed $p < .01$, ‡ indicates two-tailed $p < .001$.

[a] For mean-centered post-traumatic stress symptom severity at baseline, the model value of 0 = 50.25 (*SD* = 7.84). Baseline scores were collected four weeks prior to pre-intervention testing.

[b] Standardized combined adherence was calculated by first adding participants' total HIFT workouts and RES practices completed before subtracting the grand mean (*M* = 69.90, *SD* = 16.12). This value was then divided by the standard deviation of the grand mean. Outliers were not removed to best characterize effects on the full availability of participant data.

[c] Standardized HIFT adherence was calculated by subtracting the grand mean (*M* = 28.13, *SD* = 8.93) from participants' total HIFT workouts completed. This value was then divided by the standard deviation of the grand mean. Outliers were not removed.

[d] Standardized RES adherence was calculated by subtracting the grand mean (*M* = 41.77, *SD* = 8.71) from participants' total RES workouts completed. This value was then divided by the standard deviation of the grand mean. Outliers were not removed.

[e] For mean-centered additional workouts completed each week during the intervention, the model value of 0 = 3.57 (*SD* = 2.49). Outliers were not removed.

[f] For mean-centered additional minutes of exercise completed each week during the intervention, the model value of 0 = 238.04 (*SD* = 180.81). Outliers were not removed.

[g] For mean-centered RPE of additional workouts completed each week during the intervention, the model value of 0 = 13.49 (*SD* = 2.05). Outliers were not removed.
