# Supplementary material for: Effectiveness of a Mind–Body Intervention at Improving Mental Health and Performance Among Career Firefighters
Source: Int J Environ Res Public Health. 2025 Aug 6;22(8):1227. doi: 10.3390/ijerph22081227 (PMC12386839; doi:10.3390/ijerph22081227)
Supplement: Supplementary file 1 [file ijerph-22-01227-s001.zip › Table S1 Independent-Samples T-tests on Present and Absent Post-Testing Samples.pdf]

Table S1. Independent-samples *t*-test results for multiple outcomes between those present ($n = 20$) and absent ($n = 10$) for post-testing.

**Outcome**		***M* diff. (*SE*)**	**95% CI**	***t***	***p*[a]**	**Hedges' *g*** Point estimate	**Hedges' *g*** 95% CI
Adherence	Total HIFT Workouts Completed	-7.50 (2.80)*	[-13.81, -1.19]	-2.68	.03	-1.33	[-2.19, -0.45]
	Total RES Practices Completed	-3.27 (2.17)	[-7.72, 1.17]	-1.51	.14	-0.60	[-1.36, 0.20]
	Total MH Surveys Completed	-1.50 (0.61)*	[-2.87, -0.14]	-2.47	.02	-1.28	[-2.09, -0.46]
Additional Fitness Tracking	Additional Workouts per Week	-0.66 (0.80)	[-2.30, 0.97]	-0.83	.41	-0.34	[-1.14, 0.47]
	Additional Minutes of Exercise per Week	143.63 (84.86)*	[-44.25, 331.50]	1.69	.12	0.81	[0.01, 1.59]
	RPE Intensity of Additional Workouts	0.84 (0.83)	[-0.86, 2.55]	1.02	.32	0.40	[-0.38, 1.17]
Post-Intervention Score (Week 17)	PHQ-9	2.80 (2.76)*	[-3.80, 9.40]	1.02	.35	0.64	[-0.22, 1.49]
	PCL-C	5.34 (5.94)	[-6.90, 17.57]	0.90	.38	0.38	[-0.46, 1.22]
	CD-RISC10	-1.79 (2.54)	[-7.02, 3.45]	-0.70	.49	-0.30	[-1.14, 0.54]
	WEMWBS	-8.63 (4.28)	[-17.46, 0.20]	-2.02	.06	-0.91	[-1.82, 0.20]

Note. CD-RISC10, 10-item Connor-Davidson Resilience Scale; HIFT, high-intensity functional training; *M* diff., mean difference; MH, mental health; PCL-C, PTSD Checklist, Civilian version; *p*, *p*-value; PHQ-9, 9-item Patient Health Questionnaire; *t*, *t*-value; RES, resilience training; RPE, rate of perceived exertion according to Borg's 6-20 Rating of Perceived Exertion scale; *SE*, standard error of the mean difference; WEMWBS, Warwick-Edinburgh Mental Wellbeing Scale; 95% CI, 95% confidence interval. All *p*-values presented above are two-tailed.

* Equal of variances not assumed as indicated by a significant Levene's Test ($p < .05$). The more conservative statistic is provided above for equal variances not assumed.

[a] Raw *p*-values are presented in this column for each independent-samples *t*-test. However, a Bonferroni correction for the ten comparisons above yields a more conservative *p*-value of .005. Comparisons between groups should be weighed against this value.
