# Supplementary material for: Effectiveness of a Mind–Body Intervention at Improving Mental Health and Performance Among Career Firefighters
Source: Int J Environ Res Public Health. 2025 Aug 6;22(8):1227. doi: 10.3390/ijerph22081227 (PMC12386839; doi:10.3390/ijerph22081227)
Supplement: Supplementary file 1 [file ijerph-22-01227-s001.zip › Table S2 Self-Reported Additional Exercise Types.pdf]

Table S2. Additional exercise types reported by participants during the intervention period.

	**Major Categories (*n*)**			
	**Cardiorespiratory (205)**	**Resistance Training (186)**	**Outdoor Activities (80)**	**Mind/Body (45)**
Subcategories (*n*)	HIIT (45)	Strength/weight training (143)	Hiking (54)	Yoga (38)
	Running (45)	Functional fitness (38)	Hiking with weighted vest (14)	Stretching (7)
	Mountain biking (33)	Circuit training (4)	Backpacking (4)	
	Stationary bike (24)	Powerlifting (1)	Kayaking (3)	
	Walking (16)		Paddle boarding (2)	
	Rucking (11)		River rafting (1)	
	Jogging (8)		Rock climbing (1)	
	Spin class (8)		Wildland firefighting (1)	
	Stairs (4)			
	Swimming (4)			
	Trail running (4)			
	Road cycling (3)			
	**Major Categories (*n*; cont.)**			
	**Mixed Martial Arts (41)**	**Winter Sports (22)**	**Yard Work (12)**	**Sports (6)**
Subcategories (*n*)	Jiu Jitsu (41)	Skiing (16)	Yard work (7)	Ice Hockey (2)
		Snowboarding (4)	Snow shoveling (3)	Motocross (1)
		Cross-country skiing (2)	Wood chopping (2)	Pickleball (1)
				Tennis (1)
				Volleyball (1)

Note. Participants' responses are categorized within listed major categories and are followed by the total counts (*n*) that were reported over the intervention period in parentheses.
