# Supplementary material for: Effectiveness of a Mind–Body Intervention at Improving Mental Health and Performance Among Career Firefighters
Source: Int J Environ Res Public Health. 2025 Aug 6;22(8):1227. doi: 10.3390/ijerph22081227 (PMC12386839; doi:10.3390/ijerph22081227)
Supplement: Supplementary file 1 [file ijerph-22-01227-s001.zip › Table S3 Main effects of demographic variables on depressive symptoms centered at pre-intervention (week 4).pdf]

Table S3. Main effects of demographic variables on depressive symptoms centered at pre-intervention (week 4).

Parameter	Model 1 *B* (*SE*)	Model 2 *B* (*SE*)	Model 3 *B* (*SE*)	Model 4 *B* (*SE*)	Model 5 *B* (*SE*)	Model 6 *B* (*SE*)	Model 7 *B* (*SE*)	Model 8 *B* (*SE*)	Model 9 *B* (*SE*)	Model 10 *B* (*SE*)
**Fixed Effects**										
Intercept	3.12‡ (0.58)	3.63‡ (0.63)	3.63‡ (0.46)	3.60‡ (0.46)	3.66‡ (0.60)	2.94‡ (0.60)	3.60‡ (0.45)	3.33† (1.13)	3.91‡ (0.90)	3.55‡ (0.47)
Baseline score$_{MC}$[a]			0.76‡ (0.12)	0.74‡ (0.12)	0.76‡ (0.12)	0.71‡ (0.12)	0.72‡ (0.12)	0.75‡ (0.12)	0.76‡ (0.12)	0.76‡ (0.12)
Age$_{MC}$ (years)[b]				0.04 (0.05)						
Education[c]					-0.06 (0.78)					
Rank[d]						1.29 (0.76)				
Years of service$_{MC}$[e]							0.06 (0.05)			
Race and ethnicity[f]								0.34 (1.19)		
Relationship status[g]									-0.35 (0.96)	
Sex[h]										0.78 (1.26)
Growth		-0.08* (0.04)	-0.07 (0.04)	-0.07 (0.04)	-0.07 (0.04)	-0.07 (0.04)	-0.07 (0.04)	-0.07* (0.04)	-0.07 (0.04)	-0.07* (0.04)
**Random Effects**										
Intercept	8.71‡ (2.61)	8.83‡ (2.62)	3.12† (0.14)	3.02† (1.11)	3.12† (1.14)	2.74† (1.04)	2.85† (1.08)	3.11† (1.14)	3.10† (1.13)	3.07† (1.14)
Residual	3.87‡ (0.73)	3.60‡ (0.68)	3.58‡ (0.67)	3.58‡ (0.67)	3.58‡ (0.67)	3.58‡ (0.367)	3.58‡ (0.67)	3.58‡ (0.67)	3.58‡ (0.67)	3.58‡ (0.67)
**Pseudo R^2**										

		.0128	.4640	.4723	.4642	.4950	.4869	.4641	.4653	.4681
**Model Deviance**										
–2 log-likelihood	420.5	416.5	391.2	390.5	391.2	388.4	389.3	391.1	391.0	390.8
AIC	426.5	424.5	401.2	402.5	403.2	400.4	401.3	403.1	403.0	402.8
BIC	430.7	430.1	408.2	410.9	411.6	408.8	409.7	411.5	411.4	411.2

Note: AIC, Akaike Information Criterion; BIC, Bayesian Information Criterion; *SE*, standard error. Depressive symptom severity was measured using the 9-item Patient Health Questionnaire (PHQ-9; range = 0-27).

* indicates two-tailed $p < .05$, † indicates two-tailed $p < .01$, ‡ indicates two-tailed $p < .001$.

[a] For mean-centered depressive symptom severity at baseline, the model value of 0 = 3.43 (*SD* = 3.22). Baseline scores were collected four weeks prior to pre-intervention testing.

[b] Participants' age was centered at 39 years (*M* = 39.70, *SD* = 7.71).

[c] For education level, model values included 0 = Some college but no degree (*n* = 2) or Associate degree (*n* = 13); and 1 = Bachelor degree (*n* = 13) or Graduate degree (*n* = 2).

[d] For fire department rank, model values included 0 = Firefighter (*n* = 8) or Engineer (*n* = 6); and 1 = Captain (*n* = 10) or Battalion Chief (*n* = 6).

[e] Participants' years in the fire service was centered at 15 years (*M* = 15.43, *SD* = 8.37).

[f] Participants' responses for race and ethnicity were combined into one common model predictor. Model values included 0 = (Race: White [*n* = 1], Other [*n* = 1], Don't know [*n* = 1], or Prefer not to say [*n* = 1]; Ethnicity: Hispanic [*n* = 3] or Prefer not to say [*n* = 1]); and 1 = (Race: White [*n* = 26]; Ethnicity: Not Hispanic [*n* = 26]).

[g] For participants' relationship status, model values included 0 = Single (*n* = 3), In a relationship (*n* = 2), or Divorced (*n* = 1); and 1 = Married (*n* = 24).

[h] Model values included 0 = Male (*n* = 27), and 1 = Female (*n* = 3).
